# Supplementary material for: De novo screening of disease-resistant genes from the chromosome-level genome of rare minnow using CRISPR-cas9 random mutation
Source: Gigascience. 2021 Nov 19;10(11):giab075. doi: 10.1093/gigascience/giab075 (PMC8782236; doi:10.1093/gigascience/giab075)
Supplement: giab075_GIGA-D-21-00147_Revision_1 [file giab075_giga-d-21-00147_revision_1.pdf]

GigaScience

De novo screening of disease-resistant genes from the chromosome-level genome of rare minnow using CRISPR-cas9 random mutation

--Manuscript Draft--

**Manuscript Number:**	GIGA-D-21-00147R1
**Full Title:**	De novo screening of disease-resistant genes from the chromosome-level genome of rare minnow using CRISPR-cas9 random mutation
**Article Type:**	Data Note
**Funding Information:**	the National Natural Science Foundation of China (31972788) — Dr. Rong Huang<br>State Key Laboratory of Desert and Oasis Ecology (CN) (2019FBZ05) — Mr. Yaping Wang<br>State Key Laboratory of Desert and Oasis Ecology (2021FB11) — Mr. Yaping Wang
**Abstract:**	Background: Mutants are important for the discovery of functional genes and creation of germplasm resources. Mutant acquisition depends on the efficiency of mutation technology and screening methods. CRISPR-Cas9 technology is an efficient gene editing technology mainly used for editing a few genes or target sites, which has not been applied for the construction of random mutant libraries and for the de novo discovery of functional genes. Results: In this study, we first sequenced and assembled the chromosome-level genome of wild-type rare minnow as a susceptible model of hemorrhagic disease, obtained a 956.05 M genome sequence, assembled the sequence into 25 chromosomes, and annotated 26,861 protein-coding genes. Thereafter, CRISPR-Cas9 technology was applied to randomly mutate the whole genome of rare minnow with the conserved bases (TATAWAW and ATG) of the promoter and coding regions as the target sites. The survival rate of hemorrhagic disease in the rare minnow gradually increased from 0% (the entire wild-type population died after infection) to 38.24% (F3 generation). Finally, seven susceptible genes were identified via genome comparative analysis and cell-level verification based on the rare minnow genome. Conclusions: The results provided the genomic resources for wild-type rare minnow, and confirmed that the random mutation system designed using CRISPR-Cas9 technology in this study is simple and efficient, and is suitable for the de novo discovery of functional genes and creation of a germplasm related to quality traits .
**Corresponding Author:**	亚平 汪<br>CAS IHB: Institute of Hydrobiology Chinese Academy of Sciences<br>Wuhan, CHINA
**Corresponding Author Secondary Information:**	
**Corresponding Author's Institution:**	CAS IHB: Institute of Hydrobiology Chinese Academy of Sciences
**Corresponding Author's Secondary Institution:**	
**First Author:**	Rong Huang
**First Author Secondary Information:**	
**Order of Authors:**	Rong Huang
	Mijuan Shi
	Lifei Luo
	Cheng Yang
	Mi Ou

Powered by Editorial Manager® and ProduXion Manager® from Aries Systems Corporation

	Wanting Zhang
	Lanjie Liao
	Yongming Li
	Xiaoqin Xia
	Zuoyan Zhu
	Yaping Wang
**Order of Authors Secondary Information:**	
**Response to Reviewers:**	GIGA-D-21-00147<br>De novo screening of disease-resistant genes at the chromosome-level genome of rare minnow using CRISPR/cas9 random mutation<br>Lifei Luo; Mijuan Shi; Cheng Yang; Mi Ou; Wanting Zhang; Lanjie Liao; Yongming Li; Xiaoqin Xia; Zuoyan Zhu; Rong Huang; Yaping Wang<br>GigaScience<br><br>Dear Mr. 汪,<br>Your manuscript "De novo screening of disease-resistant genes at the chromosome-level genome of rare minnow using CRISPR/cas9 random mutation" (GIGA-D-21-00147) has been assessed by our reviewers (sorry for the delay - we needed three reviewers in this case, to cover both aspects of your paper, the CRSIPR technique and whole genome assembly).<br>Although the paper is of interest, we are unable to consider it for publication in its current form. The reviewers have raised a number of points which we believe would improve the manuscript and may allow a revised version to be published in GigaScience.<br>Their reports are below.<br>If you are able to fully address these points, we would encourage you to submit a revised manuscript to GigaScience. Once you have made the necessary corrections, please submit online at:<br>https://www.editorialmanager.com/giga/<br>Please make sure to include BUSCO analysis, using the latest version of the software. BUSCO output files should be stored on our server, for release via GigaDB in case of acceptance.<br>If you have forgotten your username or password please use the "Send Login Details" link to get your login information. For security reasons, your password will be reset.<br>Please include a point-by-point within the 'Response to Reviewers' box in the submission system. Please ensure you describe additional experiments that were carried out and include a detailed rebuttal of any criticisms or requested revisions that you disagreed with. Please also ensure that your revised manuscript conforms to the journal style, which can be found in the Instructions for Authors on the journal homepage. If the data and code has been modified in the revision process please be sure to update the public versions of this too.<br>The due date for submitting the revised version of your article is 01 Nov 2021.<br>I look forward to receiving your revised manuscript soon.<br>Best wishes,<br>Hans Zauner<br>GigaScience<br>www.gigasciencejournal.com<br>Answer: Thank you for giving us a chance to resubmit this manuscript, and also thank the reviewers for giving us constructive suggestions, which have helped us both in English and in depth to improve the quality of the paper. We have performed genome assessment using BUSCO v5.2.2 and uploaded the output files to GigaDB via FTP. At the same time, the relevant results were added to the revised manuscript and marked in blue font.<br><br>Reviewer reports:<br>Reviewer #1: In this study, "De novo screening of disease-resistant genes at the chromosome-level genome of rare minnow using CRISPR/cas9 random mutation" Luo et al. reported a chromosome-level genome of wild-type rare minnow and a new approach of mutagenesis using universal guide RNAs for CRISPR-Cas9. Compared to genome-wide CRISPR knockout (GeCKO) screening, this approach does not require

species-specific guide RNA libraries as the universal gRNA are designed to target all possible promoters or start codon regions. It demonstrated that candidate genes for disease resistance can be found using this approach. While this study presents a novel and useful approach of de novo screening of disease-resistant genes, I found some aspects that could be improved.

In page 7, in the 3rd paragraph of the Discussion, '..however, the disadvantage is that it requires sufficient candidate gene sequences.', it is true but the method in the paper also requires the whole genome sequence information to analyse the mutations (although it is not required to design gRNAs in your method).
Answer: Thank you for your careful and valuable suggestions. We agree with you. This method needs genome sequence information. We have added this point to the revised manuscript, please see the 4th paragraph of the discussion.

In page 7, in the 4th paragraph of the Discussion, could you clarify what the mutation efficiency means? For example, when you say 0.23% mutation efficiency in rare minnow does it means 0.0023 X 943.44M bp = 2.17 M bp has been mutated in individual fish?
Answer: The “mutation efficiency” here refers to the proportion of individuals with mutation characteristics in a population. This usage is inaccurate and may cause ambiguity, so we have changed it to “mutant frequency”. Please see the 4th paragraph of the discussion.

In page 12, in the 3rd paragraph, could you describe the criteria for the four levels of contribution to gene function changes?
Answer: The four levels of contribution to gene function changes were according to the snpEff definition, which are shown in the followd website: https://pcingola.github.io/SnpEff/se_inputoutput/#impact-prediction. We have added this website to the revised manuscript. Please see the 1st paragraph on page 13.

In figure 2 d, e & f, could you consider making X-axis aligned among fig. 2 d, e & f? For example, make all X axis to have 15 days post infection as maximum value or 20 dpi.
Answer: Thank you for your reminder. We have redrawn these diagrams. Please see the Fig. 2d-f in the revised manuscript.

In figure 3, what does arrow means? Could you consider improve the figure 3 and the text in the 2nd paragraph in page 6 ('There were 147,679 (139632 + 1377 + …') for better readability?
Answer: We have modified the figure 3 and the corresponding text description in order to clearly illustrate the filtering process of indels. Please see the figure 3 and the text in the 2nd paragraph on page 6 of the revised manuscript.

The viral loads were around 2 fold reduced in the knock-down (KD) grass carp cells for 7 target genes. However, 2 fold reduction in viral copy numbers is considered to be a subtle change and more importantly the viral load cannot confirm whether the viral infectivity was reduced. Analysing the infectivity of the virus produced from the KD cells by TCID50 or plaque assay together with the viral load is recommended.
Answer: Thank you for your constructive suggestions. We have supplemented experiments to analyze the infectivity of the virus produced from the KD cells by TCID50. The results showed that inhibition of the expression of these seven genes did affect the titer of GCRV. Please see the Fig. 4c in the revised manuscript.

Regarding the viral load experiment, have you checked the viral load in the resistant and susceptible minnow in vivo? If so, was there a difference in viral load between the resistant and susceptible fish?
Answer: Since we have not yet obtained 100% resistant families (All individuals can survive after infection), this means that we cannot know which individuals are resistant to disease before infection. After infection, the surviving resistant individuals are likely to have a certain immune memory or antibody level. If the surviving resistant individuals and wild individuals are infected with virus at the same time again, the real differences in viral load between the resistant and susceptible fish may not be obtained due to the different immune backgrounds of the host. I don't know whether our consideration is reasonable. Thank you.

Powered by Editorial Manager® and ProduXion Manager® from Aries Systems Corporation

I'm wondering why did you knock down the targets in grass carp cells instead of knockout? Knockout would be more suitable to validate the function of your candidate genes.
Answer: Thank you for your valuable suggestions. Because knock down can verify the gene function faster and more comprehensively, and the knock down results have proved that the genes we screened are effective. Knocking out of 23 genes is a time-consuming process. In following study, we will use knockout technology to further verify the function of candidate genes.

Could you delineate the limitation of this approach in the discussion? For examples, for species with long generation time or screening for quantitative traits, this may not be feasible and etc.
Answer: We have added relevant comments in the 4th paragraph of the discussion in the revised manuscript.

gRNAs were designed to target all protein coding genes but as the gRNA quantity for each target region is extremely low, so I think the mutations most likely happen to 'easy to target loci' which could leave numerous protein coding genes unmutated. Did you check how many genes were mutated in the founder fish before the viral challenge (to check the genome-wide mutation coverage of this 26 gRNA system)?
Answer: Thank you and we agree with you. Although this mutation method is aimed at a large number of protein coding genes, in fact, due to the limited number of gRNA, we cannot guarantee that all genes could be mutated. Because the mutation sites of different founder fish were different, and there was no suitable control population for comparison, we did not detect the mutation sites of the founder fish. However, we found that the number of possible mutation sites in disease-resistant fish was 11,668, covered by 2,441 genes, accounting for 9.09% (2,441/26,861) of the total annotated genes.

Typos or minor changes:
P6, 2nd paragraph, '..(inner ring of Fi. 3).' to be '..(inner ring of Fig. 3).'
Answer: We have modified it according to your suggestion. Please see the 2nd paragraph on page 6 in the revised manuscript.

P12, 3rd paragraph, '…without a typing result..' to be '…without a genotyping result..'.
Answer: We have modified it according to your suggestion. Please see the 1st paragraph on page 13 in the revised manuscript.

P13, 2nd paragraph, 'Genes with inhibitory effects…' to be 'siRNAs with inhibitory effects…'.
Answer: We have modified it according to your suggestion. Please see the 3rd paragraph on page 13 in the revised manuscript.

P3, 3rd paragraph, please consider use 'CRISPR-Cas9' instead of 'CRISPR/Cas9' as '-' means 'and' while '/' means 'or'.
Answer: We have revised this word in the full text according to your suggestion.

Reviewer #2: This is an interesting experiment using an innovative combination of random CRISPR/Cas9 mutagenesis with GCRV infection as a powerful selection tool to screen for disease relevant genes. Because of such a high degree of degeneracy in the gRNA sequences, it seems unlikely there would be bi-allelic mutations of any given gene in the injected embryos and thus the method would only find genes in which there is a mono-allelic affect. For the same reason, I was initially concerned that the GCRV survival phenotype would be a result from a combination of so many differ individual mutations making it difficult to isolate individual relevant genes. However, it does seem the screening methods described are sufficient to find at least some GCRV relevant genes, and thus this work is definitely useful and very worthwhile to report. I do have a few concerns and questions I hope can be addressed.

I am somewhat skeptical there was value targeting the TATA box considering only ~20% of promoters contain this element. Was it recorded what percentage of the 23 hemorrhagic disease associated loci came from TATAWAW targeting gRNAs in contrast to ATG targeting gRNAs? This information should be included and discussed in the manuscript.

Powered by Editorial Manager® and ProduXion Manager® from Aries Systems Corporation

	Answer: Thank you for your constructive suggestions. We analyzed the TATAWAW and ATG targets close to 23 hemorrhagic disease-associated loci. It was found that the targets of 22 loci in 23 were ATG, and the targets of one site could not be determined because both TATAWAW and ATG targets were nearby (within 20bp). The proportion of mutations from ATG targets in 23 loci was close to 100%. This result was included and discussed, please see the 2nd paragraph on page 6 and the 4th paragraph on page 7 in the revised manuscript.<br><br>In the "Functional verification of susceptible genes related to hemorrhagic disease" section of the results, the 23 candidate genes were narrowed down to 9. The wording was not clear, you wrote "This indicated that the siRNA of the nine genes had a significant inhibitory effect". I'm assuming you meant, This indicated that nine siRNAs had a significant inhibitory effect".<br>Answer: We have changed it to "The results indicated that nine siRNAs had a significant inhibitory effect ($p < 0.05$)" according to your suggestion. Please see the "Functional verification of susceptible genes related to hemorrhagic disease" section of the results.<br><br>In the methods section, it is not clear why every gRNA sequence was started with an NNR. This should be clarified.<br>Answer: Thank you for your reminder. Sorry, this is a low-level mistake we made, it should be GGR. Due to the preference of T7 promoter (used to make gRNAs) for transcription initiation sites, many early studies suggested that transcription should better start with GG, and the third base should better be A or G (Hwang ER et al. 2013; https://www.docin.com/p-915537211.html). In its current implementation, our gRNA-Cas9 system described above can theoretically target the sequence with the form 5'-GGR-N0-10-TATAWAW-N10-0-NGG-3' and 5'-GGR-N0-14-ATG-N14-0-NGG-3'.<br>References:<br>1. Hwang WY, Fu Y, Reyon D, et al. Efficient In Vivo Genome Editing Using RNA-Guided Nucleases. Nature Biotechnology, 2013, 31(3):227-229.<br>2. "Cas9 protocol_v11_130216" in https://www.docin.com/p-915537211.html<br><br>As far as functional validation, it seems micro-injection of single gene specific gRNAs targeting the 23 candidate genes from the initial screen would have been most appropriate and should be done in addition to or in place of the siRNA treated GCO cell line validation. What was the rationale behind taking only this latter approach? This rationale should be outlined in the manuscript to better justify the experimental design.<br>Answer: Thank you for your valuable suggestions. The purpose of gene knockdown in this study is to quickly and effectively verify the screened candidate genes. If knockout is done in rare minnow in vivo, each knockout family needs to be established, it requires at least two generations and more than 8 months. On the other hand, there is no mature CRISPR-cas9 knockout system suitable for fish cell line at present, so the knockout experiment at cell level may not be carried out stably and effectively. In view of the above considerations, this study adopted the experimental design of knockdown of GCO cell line, which can ensure that we can quickly and effectively preliminarily verify the function of candidate genes. We will later use gRNA mediated knockout to deeply analyze the function of candidate genes. We have outlined the rationale in the revised manuscript, please see the 2nd paragraph on page 13.<br><br>Reviewer #3: This paper entitled as 'De novo screening of disease-resistant genes at the chromosome-level genome of rare minnow using CRISPR/cas9 random mutation' has shown a novel chromosome-level genome of rare minnow. The authors performed a series of analyses, including genome assembly, annotation, evolution and gene editing. The methods used in this paper are really solid and common.<br><br>However, the most significant detect is why this fish is a better gene editing model than zebrafish, which is not clearly described in this study.<br>Answer: Thank you for your careful review. As a relatively new model fish, rare minnow is not better than zebrafish in every aspect. The reason why the rare minnow was selected as a gene editing model in this study is that it is very sensitive to GCRV virus. The mortality of wild-type rare minnow infected with this virus is 100%, which provides us with an excellent screening method for GCRV resistance, that is, the individuals that can survive after infection may be the individuals with successful mutation. This view was supplementary descripted and explained in the 1st and 5th paragraph of the

introduction in the revised manuscript.

On the other hand, the genome assembly has not been evaluated by BUSCO.
Answer: We have performed genome assessment using BUSCO v5.2.2. Please see the “Genome assembly and annotation” section of the Results and the “Genome sequencing and assembly” section of the Methods.

The English language of this paper needs a large of revision.
Answer: We have submitted the revised manuscript to a professional company for language modification and polishing.

In Abstract, the assembled chromosome number should be clarified in this section.
Answer: Thank you for your reminder. We have added the number of assembled chromosomes in this section. Please see the abstract of the revised manuscript.

In Introduction, 'It is worth noting that they are more sensitive to organic pollutants and pathogenic microorganisms than zebrafish (Danio rerio) and medaka (Oryzias latipes).' This sentence should be more clarified why this species is more sensitive. The related reference also should be complemented in this sentence.
Answer: This sentence we wrote was not rigorous, so we have revised it and added references. Please see the 1st paragraph of the introduction in the revised manuscript.

"Traditional physical and chemical mutagenesis methods mainly cause genomic point mutations [4-6], which cannot be distinguished from natural SNP mutations, which leads to considerable difficulties while performing comparative analysis of the subsequent functional genomes." This sentence should be revised, two 'which' are indeed strange.
Answer: Thank you for your suggestion. We have changed this sentence to “Traditional physical and chemical mutagenesis methods mainly cause genomic point mutations [4-6], which cannot be distinguished from natural SNP mutations, leading to considerable difficulties while performing comparative analysis of the subsequent functional genomes”. Please see the 3rd paragraph of the introduction in the revised manuscript.

'The traditional transposon mutation has' revised to 'The traditional transposon mutations have'.
Answer: We have modified it according to your suggestion. Please see the 3rd paragraph of the introduction in the revised manuscript.

'On one hand, the cost of designing a large number of sgRNAs is high; on the other hand, it is only suitable for the construction of a mutant library of known candidate genes.' should be revised.
Answer: We have changed this sentence to “In this way, a large number of sgRNAs needed be designed at a high cost, and it is only suitable for the construction of a mutant library with known candidate genes” Please see the 4th paragraph of the introduction in the revised manuscript.

'Therefore, the genome of the rare minnow was deemed to be a simple one.' What's real meaning of the 'simple one'?
Answer: "Simple one" means that the repetitive sequence proportion and heterozygosity of the genome are not high, and the genome can be easily sequenced and assembled. For ease of understanding, we have changed this sentence to “The results showed that the genome of rare minnow was a simple genome rather than a complex one”. Please see the “Genome assembly and annotation” section of the Results.

'the N50 was 21,231 bp' should be keep two decimals.
Answer: We have changed it to “the N50 was 21,231.00 bp”. Please see the 2nd paragraph on page 4.

'181,735,272 pairs of unique mapped read pairs were obtained' revise to '181,735,272 pairs of unique mapped reads were obtained'.
Answer: We have modified it according to your suggestion. Please see the 3rd paragraph on page 4.

	'a total of 36,387 mRNA encoding proteins were annotated, corresponding to 26,861 genes.' Why 26,861 genes were related to 36387 mRNA? This set contains alternative splicing results?<br>Answer: Yes, there were 4,957 genes with alternative splicing transcripts. We have supplemented the relevant description, please see the “Genome assembly and annotation” section of the Results.<br><br>No BUSCO evaluation result for the genome assembly.<br>Answer: We have performed genome assessment using BUSCO v5.2.2. Please see the "Genome assembly and annotation" section of the Results and the "Genome sequencing and assembly" section of the Methods.<br><br>Suggest the authors to draw a figure to show the random genome editing pipeline more clear.<br>Answer: We have drawn a figure to show the random genome editing pipeline, please see the Additional File 8 in the revised manuscript.<br><br>'Evolutionary analysis of the genome' should be 'Evolutionary analysis of the genomes'<br>Answer: We have modified it according to your suggestion. Please see the title lines on page 4 and 10.<br><br>'the 351 individuals of the control group (wild-type) all died' remove the second 'all'.<br>Answer: We have modified it according to your suggestion. Please see the 3rd paragraph on page 5.<br><br>'The cumulative mortality curves was drawn' should 'were drawn'.<br>Answer: We have modified it according to your suggestion. Please see the 1st paragraph on page 12.<br><br>'Three wild-type females' should be 'Three wild-type female'.<br>Answer: We have modified it according to your suggestion. Please see the 2nd paragraph on page 12.<br><br>'in the same position in C.' changes to 'in the same position in C group'.<br>Answer: We have modified it according to your suggestion. Please see the 2nd paragraph on page 6.<br><br>All the software should be added the version and RRID in Gigascience journal.<br>Answer: Thank you and we have added the RRIDs of all software used in our manuscript.
**Additional Information:**	
**Question**	**Response**
Are you submitting this manuscript to a special series or article collection?	No
**Experimental design and statistics**<br><br>Full details of the experimental design and statistical methods used should be given in the Methods section, as detailed in our Minimum Standards Reporting Checklist. Information essential to interpreting the data presented should be made available in the figure legends.<br><br>Have you included all the information requested in your manuscript?	Yes

**Resources**<br><br>A description of all resources used, including antibodies, cell lines, animals and software tools, with enough information to allow them to be uniquely identified, should be included in the Methods section. Authors are strongly encouraged to cite Research Resource Identifiers (RRIDs) for antibodies, model organisms and tools, where possible.<br><br>Have you included the information requested as detailed in our Minimum Standards Reporting Checklist?	Yes
**Availability of data and materials**<br><br>All datasets and code on which the conclusions of the paper rely must be either included in your submission or deposited in publicly available repositories (where available and ethically appropriate), referencing such data using a unique identifier in the references and in the “Availability of Data and Materials” section of your manuscript.<br><br>Have you have met the above requirement as detailed in our Minimum Standards Reporting Checklist?	Yes

Manuscript
Click here to access/download;Manuscript;Revised manuscript.docx

De novo screening of disease-resistant genes from the chromosome-level genome of rare minnow using CRISPR-cas9 random mutation

Rong Huang[1, †, *], Mijuan Shi[1, †], Lifei Luo[1], Cheng Yang[1], Mi Ou[1], Wanting Zhang[1], Lanjie Liao[1], Yongming Li[1], Xiao-Qin Xia[1], Zuoyan Zhu[1], Yaping Wang[1, 2, *]

[1] State Key Laboratory of Freshwater Ecology and Biotechnology, Institute of Hydrobiology, Chinese Academy of Sciences, Wuhan 430072, China. shimijuan@ihb.ac.cn (M.S.); luolifei145@163.com (L.F.L.); yc_plan@163.com (C.Y.); 503095653@qq.com (M.O.); zhangwanting@ihb.ac.cn (W.Z.); liaolj@ihb.ac.cn (L.J.L.); liym@ihb.ac.cn (Y.L.); xqxia@ihb.ac.cn (X.X.); zyzhu@ihb.ac.cn (Z.Z.)

[2] Innovative Academy of Seed Design, Chinese Academy of Sciences, Beijing 100101, China

[*] Corresponding authors.

E-mail eddresses: huangrong@ihb.ac.cn (R.H.), wangyp@ihb.ac.cn (Y.W.)

Tel.: +86-027-68780081; Fax: +86-027-68780123

[†] These authors contributed equally to this work.

Abstract

Background: Mutants are important for the discovery of functional genes and creation of germplasm resources. Mutant acquisition depends on the efficiency of mutation technology and screening methods. CRISPR-Cas9 technology is an efficient gene editing technology mainly used for editing a few genes or target sites, which has not been applied for the construction of random mutant libraries and for the *de novo* discovery of functional genes. **Results:** In this study, we first sequenced and assembled the chromosome-level genome of wild-type rare minnow as a susceptible model of hemorrhagic disease, obtained a 956.05 M genome sequence, assembled the sequence into 25 chromosomes, and annotated 26,861 protein-coding genes. Thereafter, CRISPR-Cas9 technology was applied to randomly mutate the whole genome of rare minnow with the conserved bases (TATAWAW and ATG) of the promoter and coding regions as the target sites. The survival rate of hemorrhagic disease in the rare minnow gradually increased from 0% (the entire wild-type population died after infection) to 38.24% (F3 generation). Finally, seven susceptible genes were identified via genome comparative analysis and cell-level verification based on the rare minnow genome. **Conclusions:** The results provided the genomic resources for wild-type rare minnow, and confirmed that the random mutation system designed using CRISPR-Cas9 technology in this study is simple and efficient, and is suitable for the *de novo* discovery of functional genes and creation of a germplasm related to quality traits.

Keywords**:** Rare minnow; Genome; CRISPR-Cas9; Mutant; Germplasm resource

Introduction

Rare minnow (*Gobiocypris rarus*) belongs to the order Cypriniformes and family Cyprinidae, and it has the advantages of a small body, fast reproduction, and easy feeding. It is more sensitive to some pollutants compared to zebrafish (*Danio rerio*) and medaka (*Oryzias latipes*). For example, the sensitivity of rare minnow to 17α-ethinylestradiol and pentachlorophenol is higher than that of zebrafish, and its sensitivity to ethinylestradiol is higher than that of medaka [1-3]. Therefore, it has been widely employed in genetics, physiology, biological monitoring, toxicity testing, and other fields [4].

The mortality rate of rare minnow infected with grass carp (*Ctenopharyngodon idellus*) reovirus (GCRV) is 100% [5]. Grass carp, which also belongs to the family Cyprinidae, is one of the most important

freshwater fishes worldwide. The mortality of grass carp hemorrhagic disease caused by GCRV infection is more than 80% [6], which poses a great threat to the development of the aquaculture industry. Rare minnow, similar to grass carp, is highly sensitive to GCRV, which makes it an ideal model for studying grass carp hemorrhagic disease and exploring germplasm resources.

Research on efficient mutation methods is a prerequisite for constructing an ideal animal model. Traditional physical and chemical mutagenesis methods mainly cause genomic point mutations [7-9], which cannot be distinguished from natural SNP mutations, leading to considerable difficulties when performing comparative analyses of the subsequent functional genomes. Traditional transposon mutations have a strong selectivity for the mutation region of the receptor genome, and they are unable to achieve random mutations for all genes [10, 11]. Efficient and easy-to-detect mutation methods are important for obtaining mutants and for exploring new germplasm resources.

CRISPR-Cas9 technology is an efficient gene editing technology mainly used for editing a few genes or target sites [12, 13]. It is also used to study mutant libraries. In previous research on human cells and rice, the main way to construct a mutant library was to design sgRNA of all candidate genes, then mix all sgRNAs, and select target mutants after knockout [14-17]. In this way, a large number of sgRNAs needed be designed at a high cost, and it is only suitable for the construction of a mutant library with known candidate genes. To date, efficient CRISPR-Cas9 technology has not been applied for the construction of random mutant libraries and for the *de novo* discovery of functional genes.

All wild-type rare minnows die after being infected with GCRV, which provides us with an excellent mutant screening material for GCRV resistance. That is, the individual who can survive after infection is likely to be an individual with successful mutations. In this study, we assembled a high-quality genome of rare minnow, and then used CRISPR-Cas9 technology to randomly mutate the complete genome of the rare minnow and obtained a mutant population with GCRV resistance traits. Next, we obtained seven hemorrhagic disease-susceptible genes via genome comparative analysis and experimental verification. The results not only provided genomic resources for research on rare minnow, but also facilitated the establishment of a simple and feasible method for random genomic mutations, which are suitable for the exploration of functional genes and new germplasm resources.

Results

Genome assembly and annotation

To initially evaluate the genome of rare minnow, we obtained 124.20 G raw data and 121.11 G clean data after routine filtering. Based on the K-mer (K = 21) analysis method, the genome size was estimated to be 943.44 M, the heterozygosity rate was 0.41%, and the repetition rate was 35.82%. The results showed that the genome of rare minnow is a simple genome rather than a complex one.

After filtering the PacBio data, 106.88 G subreads were obtained. The mean length of the subreads was 13,088.61 bp, and the N50 was 21,231.00 bp. After the subreads data were self-corrected, the genome was assembled into a size of 960.27 M, consisting of 858 contigs with an N50 of 5.46 M. Using the 121.11 G NGS data obtained previously, the assembled genome was corrected again, and the final size of the corrected genome was 959.10 M and the contig N50 was 5.46 M.

We then obtained 103.47 G clean data from Hi-C library sequencing. After filtering and evaluating with HIC-pro, 181,735,272 pairs of uniquely mapped reads were obtained, of which 123,097,523 pairs were valid interaction pairs, accounting for 67.73% of the total number of pairs. Based on valid interaction pairs, a 924.69 M sequence composed of 345 contigs was assembled into 25 chromosomes, accounting for 96.41% of the total sequence length (Table 1). The completeness of the genome was 90.20% based on the BUSCO evaluation. A heat map describing the contact matrix was constructed to evaluate the accuracy of the Hi-C assembly (Fig. 1a). The interaction signals obtained from the heat map could help to clearly distinguish the 25 chromosomes, indicating that the assembly effect of the genome was very good.

We annotated 43.14% of the rare minnow genome as repetitive sequences (Additional File 1: Table). In addition, 36,387 mRNAs were annotated, corresponding to 26,861 genes, of which 4,957 genes had alternative splicing transcripts. The average length of the longest CDS of all genes was 1.82 K, which was close to the average length of zebrafish and higher than that of grass carp and blunt snout bream (*Megalobrama amblycephala*) (Additional File 2: Table).

Evolutionary analysis of the genomes

Through cluster analysis of gene families of 12 species, 23,640 gene families were obtained, among which 2,867 were shared gene families, 15,689 were shared genes, and 1,097 were single-copy gene families. A

phylogenetic tree was constructed using all single-copy gene families (Fig. 1b). Fig. 1b shows that four Cyprinidae species were clustered into one branch; the differentiation time of rare minnow and grass carp was 33.89 MYA (Fig. 1b).

Collinearity analysis showed that 18,968 similar genes were located in 97 superconsigs of grass carp. The linkage groups of the 97 supercontigs of grass carp were mapped to the genome of the rare minnow (Fig. 1c). Chromosomes 1 and 21 (LG1 and LG21) of rare minnow correspond to LG13 of grass carp, and the degree of gene collinearity of the two species was very high (Fig. 1c).

Anti-hemorrhagic model of rare minnow

Twenty-six sgRNAs were mixed with Cas9 protein and injected into approximately 8,000 single-cell embryos. Finally, 3,126 two-month-old P0 mutants were obtained. Among them, 3,000 were used in the GCRV infection experiment. The results showed that 2,993 died and seven survived, with a survival rate of 0.23%, conversely, all the 351 individuals of the control group (wild-type) died, demonstrating a survival rate of 0% (Fig. 2a). During the course of the disease, the dead individuals in the mutation and control groups exhibited a red body surface, showing obvious hemorrhagic symptoms (Fig. 2b).

To eliminate the difference in survival rate caused by experimental errors, two F1 families (F1-1 and F1-2) were obtained by lateral-crossing two surviving males from the P0 generation with wild-type females. The survival rates of F1-1 and F1-2 were 1.25% and 1.89%, respectively, and F2–F3 generation families were obtained by self-crossing, infection, and reproduction. The survival rates of the four F2 generation families (F2-1, F2-2, F2-3, and F2-4) were 3.33%, 4.85%, 17.02%, and 23.08%, respectively. Infection experiments of seven F3 families (F3-1, F3-2, F3-3, F3-4, F3-5, F3-6, and F3-7) showed that the survival rates of the F3-6 and F3-7 families were 31.37% and 38.24% higher than those of the F2-4 family (the parent source of F3 families) (Fig. 2c).

During the GCRV infection, daily deaths in the F1 to F3 generation mutant groups and the control group were counted and cumulative mortality curves were established (Fig. 2d–f). As shown in Fig. 2d, individuals in both the F1-2 family and the control group began to die as early as 5 days post-infection (dpi), while those in the F1-1 family began to die at 7 dpi. In four GCRV-infected F2 families, the mortality of the four families was higher than that of the control group at 6 dpi, but was lower after 8 dpi. In addition, the

duration of death of the F2 families was prolonged by 3-4 days compared with that in the control group (Fig. 2e). Among the seven F3 generation families, two families (F3-1 and F3-2) died faster than the control at 5 and 6 dpi, but the death rate of all mutant families was lower than that of the control after 7 dpi. The duration of deaths in the F3 mutant families was prolonged by 3-10 days compared to that in the control group (Fig. 2f). Overall, compared to the control, F1, F2, and F3 mutant families exhibited delayed death induced by GCRV infection.

Screening of candidate indel loci related to hemorrhagic disease

The indel loci and genotypes of 11 datasets (C, S1, L7, T1, T2, T3, and P1–P5) were analyzed using GATK v4.1.1.0. The genotypes of the indels in the T1, T2, and T3 groups were compared with those in the same position in the control groups (C, S1 and L7), and represented by the letters T, N, and F (Fig. 3). These genotypes were then divided into four grades: high, moderate, low, and modified based on the contribution of these loci to gene function changes. There were 147,679 (139,632 + 1,377 + 1,971 + 1,622 + 931 + 1,125 + 1,021) indels in TTT, two T + one N, and one T + two N types (outer ring of Fig. 3). Furthermore, 147,679 loci in five F1 parents (P1-P5) were genotyped, and 11,668 loci with new genotypes (F0) were identified (inner ring of Fig. 3). Combined with the contribution of sites to gene function change, the contributions of 23 loci were high among the 11,668 loci (Additional File 3: Table). These 23 loci were associated with hemorrhagic diseases. The TATAWAW and ATG targets closest to these 23 loci were analyzed. The targets of 22 loci were found to be ATG, and the targets of one site could not be determined because both TATAWAW and ATG targets were nearby (within 20 bp) (Additional File 4: Table).

Functional verification of susceptible genes related to hemorrhagic disease

According to the genome annotation information of rare minnow, 20 genes containing 23 loci related to hemorrhagic disease were identified (Additional File 5: Table). By comparing 20 genes of rare minnow with annotation information of the grass carp genome, 23 homologous genes in grass carp were obtained (Additional File 6: Table). siRNAs and specific primers for 23 grass carp genes were designed, and the sequences are shown in Additional File 7. After the siRNAs were transfected into GCO cells, the relative expression level of each target gene at 48 h post-transfection in the siRNA-transfected cells was normalized

to the expression level of the target gene at 0 h. The results indicated that nine siRNAs had a significant inhibitory effect ($p < 0.05$) (Fig. 4a). To study the effects of siRNA knockdown on GCRV infection, these nine siRNAs were transfected into GCO cells and infected with GCRV. RT-qPCR analysis showed that transfection of seven siRNAs significantly reduced the copy number of GCRV in GCO cells at 32 h post-transfection, compared with that in the NC group ($p < 0.05$) (Fig. 4b). Further, the titer of GCRV contained in GCO cells transfected with the seven siRNAs was detected. It was showed that the titer decreased significantly in the GCO cell groups treated with the seven siRNAs ($p < 0.05$) (Fig. 4c). These results suggest that these seven genes are indeed susceptible to GCRV.

Discussion

In this study, the genome sequence and annotation information of rare minnow were obtained, providing a high-quality genome analysis platform for research and use in more fields.

In addition, we established a method for constructing a genome-wide random mutant library via the special application of CRISPR-Cas9 using rare minnow as a hemorrhagic disease-susceptible model (Additional File 8: Figure). This method has a wide mutation range, low cost, and high efficiency and is suitable for functional genomics research and for creation of germplasm resources related to quality traits.

To date, some studies have used CRISPR-Cas9 technology to construct a mutant library of human cells and rice [14-17]. They designed sgRNA within the range of existing candidate genes. The advantage of this strategy is that it is helpful in detecting mutation sites; however, the disadvantage is that it requires sufficient candidate gene sequences. If there were no expected trait-related genes among the candidate genes, the expected mutant could not be obtained. The target sites in this study were designed based on the conserved bases of the gene promoter and coding region (TATAWAW and ATG) (Fig. 5), which can theoretically cover the functional region of all genes in the genome, thus increasing the abundance of mutation libraries and greatly improving the possibility of obtaining target trait mutants. In addition, the method established in this study only requires the synthesis of 26 sgRNAs, and mutants can be obtained using efficient screening methods. However, it should be noted that 23 hemorrhagic disease-associated loci were almost all produced by ATG targets (Additional File 4: Table). This may be related to the fact that only a few gene promoters contain TATA boxes. Previous studies have found that 23.85% of eukaryotic

promoter sequences contain TATA boxes, and approximately 20% of yeast genes contain a TATA box [18, 19]. Future studies are expected to consider only ATG as a mutation target.

Many studies have been conducted to construct plant mutant libraries by physical and chemical mutagenesis, with mutant frequency between 0.031% and 9.3% [20-23]. The efficiency of *Arabidopsis thaliana* mutants obtained through transposon mutagenesis was 0.091% and 1% [24, 25]. In animals, the chemical mutagen ethyl nitrosouria (ENU) is mainly used in relevant studies in some species, such as *Caenorhabditis elegans* [26, 27], zebrafish [28, 29], mouse (*Mus musculus*) [30, 31], grass carp [32] and pig (*Sus scrofa*) [33], and the mutant frequency is generally not more than 0.03%. Compared with existing studies, the mutant frequency of this method (0.23%) is similar to that in plants, but approximately 10 times higher than that in animals. In addition, another important reason why we successfully obtained resistant mutants by this method is that the selected traits were quality traits. The entire wild-type population of rare minnow died after hemorrhagic disease, individuals who survived after infection were mutated individuals, who could be easily and efficiently identified. However, it must be pointed out that this method requires whole genome sequence information and may not be feasible in the screening of quantitative traits or some species with long generation times.

Methods

Sources of experimental fish, viruses, and cells

Rare minnow samples were collected from the Liusha River, Hanyuan County, Sichuan Province, China, by the ichthyology laboratory at the Institute of Hydrobiology, Chinese Academy of Sciences. GCRVs were isolated and preserved in our laboratory. Grass carp ovary (GCO) cells were presented by Li Shun, Associate Professor at the Institute of Hydrobiology, Chinese Academy of Sciences. *Ctenopharyngodon idellus* kidney (CIK) cells were purchased from China Center for Type Culture Collection (CCTCC).

Experiments involving rare minnows in this study were carried out in accordance with the Guide for the Care and Use of Laboratory Animals (Ministry of Science and Technology of China, 2006), and the protocol was approved by the Committee of the Institute of Hydrobiology, Chinese Academy of Sciences. The reference number obtained was Y9110306.

Genome sequencing and assembly

A sexually mature female rare minnow was selected for this study. Part of the muscle tissue was frozen in liquid nitrogen and genomic DNA was extracted from the other part. The cetyltrimethylammonium bromide method was used to extract DNA. Next generation sequencing (NGS) was performed on an Illumina HiSeq X Ten platform using paired-end reads (PE) of 150 bp, and the sequencing fragments were 350 ± 50 bp. After conventional filtering, a K-mer frequency distribution map was drawn based on the K-mer (K = 21) analysis method and genome size, heterozygosity, and repetition rate were evaluated.

The PacBio Sequel system was used for third-generation sequencing (TGS). Subreads were obtained using signal-to-noise ratio (SNR) filtering. After using Canu v1.9 (Canu, RRID:SCR_015880) [34] to self-correct subreads, WTDBG v1.2.8 (WTDBG, RRID:SCR_017225) [35] was used for sequence assembly. Based on previous NGS data used for genome evaluation, the assembled genome sequence was corrected using Pilon v1.23 (Pilon, RRID:SCR_014731) [36].

The muscle tissue cryopreserved in liquid nitrogen was fixed and crosslinked with formaldehyde, and a Hi-C library was constructed. NGS was performed using an Illumina HiSeq X Ten platform. Clean data were obtained after routine filtration and compared with assembled genome sequences. The comparison results were filtered using HIC-Pro v2.11.1 (HIC-Pro, RRID:SCR_017643) [37] to obtain valid interaction pairs. Based on valid interaction pairs, the genome assembled in the previous step was divided, sorted, and oriented using LACHESIS (LACHESIS, RRID:SCR_017644) [38], and the assembly sequence at the chromosome level was obtained. The completeness of the genome was evaluated through BUSCO v5.2.2 (BUSCO, RRID:SCR_015008) [39] using the gene set of vertebrata_obd10. Then the number of Hi-C read pairs covering any two bins was used as the intensity signal of the interaction between the two bins, and a heat map was drawn to evaluate the Hi-C assembly results.

Genome annotation

Genome annotation was performed in two parts: repetitive sequence annotation and coding gene annotation. RepeatModeler v1.0.11 (RepeatModeler, RRID:SCR_015027) was used to construct a repetitive sequence library of the genome, and RepeatMasker v4.0.9 (RepeatMasker, RRID:SCR_012954) was used to mark the repetitive sequences based on the repetitive sequence library; the parameter was - now - div 20 - GC 39

[40]. Finally, previous results were further annotated using the existing repeat sequences of rare minnow in the Repbase database (Repbase, RRID:SCR_021169); the parameter was - now - div 20 - GC 39 [41].

The annotation of the coding genes integrated the results of *ab initio* gene prediction, protein sequence alignment, and transcriptional assembly. For *ab initio* gene prediction, AUGUSTUS v3.3.3 (AUGUSTUS, RRID:SCR_008417) [42], GlimmerHMM v3.0.4 (GlimmerHMM, RRID:SCR_002654) [43], geneID v1.4 (geneID, RRID:SCR_021639) [44], and SNAP v2006-07-28 (SNAP, RRID:SCR_002127) [45] were used. Whole genome protein sequences of the following related species—common carp (*Cyprinus carpio*), goldfish (*Carassius auratus*), and zebrafish (*Danio rerio*)—were used for homologous protein sequence alignment prediction using Gemoma v1.6.4 (Gemoma, RRID:SCR_017646) [46, 47]. Two strategies were employed for transcriptional assembly: with a reference genome and without a reference genome. The strategy with reference genome involved using hisat2 v2.1.0 (hisat2, RRID:SCR_015530) [48] for alignment and StringTie v1.3.5 (StringTie, RRID:SCR_016323) [49] for assembly. The strategy without the reference genome involved the assembly of Trinity v2.8.5 (Trinity, RRID:SCR_013048) [50]. The transcripts from the two sources were processed using the PASA pipeline (PASA, RRID:SCR_014656) [51], including sequence filtering and realignment analysis. Finally, the results of the three sources were evaluated with EVM (EVM, RRID:SCR_014659) [52] to obtain the gene coding regions, and the untranslated region of the gene was annotated using the PASA pipeline and transcriptome data. In AUGUSTUS, "zebrafish" was selected as the training set for prediction, and default parameters were used for all other software.

Evolutionary analysis of the genomes

From amphibians to mammals, 12 species (including rare minnow) were collected. Using Orthofinder v2.4.0 (Orthofinder, RRID:SCR_017118) [53], the protein sequences of the 12 species were classified (the DIAMOND alignment program was used, with an e-value of 0.001), and the gene families obtained were annotated using the PANTHER database (PANTHER, RRID:SCR_004869) [54] to obtain shared gene families, shared genes, and single-copy gene families among species. The obtained single-copy gene family was sorted by MAFFT v7.471 (MAFFT, RRID:SCR_011811) [55], and a phylogenetic tree was constructed using RAxML-NG v0.9.0 (RAxML-NG, RRID:SCR_006086) [56] and the maximum likelihood (ML)

method, for which the number of bootstraps was set to 1,000. Combined with fossil evidence, r8s v1.81 (r8s, RRID:SCR_021161) (https://sourceforge.net/projects/r8s/) was used to construct a phylogenetic tree with divergence time.

Using the CAFE v4.2 (CAFÉ, RRID:SCR_018924) [57] and the results of the phylogenetic tree with divergence time and gene family clustering, we estimated the number of gene family members in the ancestors of the four Cyprinidae fish species using the birth mortality model, and predicted the contraction and expansion of the gene family of the four Cyprinidae fish species relative to their ancestors (the criterion for contraction and expansion was $p < 0.05$).

Because the grass carp genome is at the supercontig level, 99 large supercontigs attached by a published genetic linkage map of grass carp were used for collinearity analysis [58]. JCVI v0.18 (JCVI, RRID:SCR_021641) [59] was used to perform protein sequence alignment between rare minnow and grass carp. Finally, a collinearity graph was drawn using Circos v0.69 (Circos, RRID:SCR_011798) (http://circos.ca/).

Establishment of an anti-hemorrhagic disease model

The promoter and coding regions were selected as the main mutation regions. While designing the mutation target site, among the 20 bases starting from GGR (requirements of T7 promoter, R for A/G), the conserved sequence TATAWAW (W for A/T) in the TATA frame and the start codon ATG were gradually shifted backward, and N was used as a supplement. The 26 primers upstream of the target site are shown in Fig. 5, and the primer downstream of the target site was AAAAAAAGCACCGACTCGGTGCCACT. After PCR amplification using the pMD-19T-gRNA plasmid as a template, 26 sgRNAs were transcribed using a TranscriptAid T7 High Yield Transcription Kit (Thermo Scientific, USA).

Twenty-six sgRNAs were mixed with Cas9 protein (Invitrogen, USA) at final concentrations of 400 ng/µL and 100 ng/µL. Each sgRNA was injected into approximately 300 rare minnow embryos, which constituted the P0 generation. At 2 months of age, a high-salt invasion method was used for GCRV infection. The method was as follows: the fish were soaked in 6% NaCl solution for 2 min and then quickly transferred to GCRV suspension (virus titer: 2.75×10^8 $TCID_{50}$/mL) for 30 min. The wild-type mixed population used as a control group was infected in the same manner. The number of dead fish in each group

was recorded daily.

From the surviving individuals of the P0 generation, male individuals were selected and lateral-crossed with wild-type female individuals to obtain F1 full-sib families. GCRV infection was performed at 2 months of age. The surviving individuals in an F1 full-sib family with the highest survival rate were self-crossed to construct F2 full sib families. The F3 generation was obtained by self-crossing in the same way and was infected with GCRV. The wild-type mixed population was used as a control group for infection. The number of deaths in the F1-F3 population and the wild-type population were counted every day after infection, those individuals who did not die for two consecutive weeks were termed survival individuals. Cumulative mortality curves were drawn, and the survival rate of each family was calculated.

Screening of candidate indels associated with hemorrhagic disease

Three surviving individuals were randomly selected from three families (F2-2, F2-3, and F2-4) with high disease-resistance in the F2 generation. Three wild-type female and three wild-type male individuals were selected. Genomic DNA was extracted from 15 fish using the high-salt method. Sequencing libraries T1, T2, and T3 were constructed by mixing the DNA of three fish in F2-2, F2-3, and F2-4, and sequencing library C was constructed by mixing the DNA of six wild-type individuals. The inserted fragment size was 350 ± 50 bp, and NGS was performed on the BGI MGISEQ-2000 platform with a PE 150. Five parents (F1 survival mutant P1-P5) of the F2 families were sequenced in the same manner. In addition, the NGS data (S1 and L7) of the two groups of wild-type were collected from our lab to increase the information richness of the control group. S1 was obtained from a wild-type female and a wild-type male mixed sample, and L7 was from a wild-type male sample.

Clean data were obtained by filtering the raw data of all samples. Using Bowtie2 v2.3.5 (Bowtie2, RRID:SCR_005476) [60], 11 datasets were compared with the reference genome of rare minnow assembled above. Then, the HaplotypeCaller of GATK v4.1.1.0 (GATK, RRID:SCR_001876) [61] was used for indel calling. Library C had six mixed samples, and the parameter was set to -- sample ploidy 12; T1, T2, and T3 had three mixed samples, and the parameter was set to -- sample ploidy 6. The indel filters of all samples were hard filtered with QD < 2, FS > 100, read position Mann –Whitney Rank-Sum < 20, and SOR > 10. Finally, VCF files were used to record the indel loci and genotypes of 11 datasets; snpEff

(snpEff , RRID:SCR_005191) (http://snpeff.sourceforge.net/) was used to annotate the VCF files.

The genotypes of each indel locus in three samples (C, S1, and L7) were combined as controls and compared with corresponding indels in eight samples (T1, T2, T3, and P1–P5). Among the eight samples, the locus with the new genotype was recorded as “T”, the locus without a genotyping result was recorded as “N”, and the locus with a genotyping result but without a new genotype was recorded as “F”. The contribution of these loci to gene function changes was used to distinguish the SnpEff annotation results, which can be divided into four levels: high, moderate, low, and modified (https://pcingola.github.io/SnpEff/se_inputoutput/#impact-prediction). Next, there were three steps in the screening process: the first step was to screen the loci that were not “F” type in T1, T2 and T3; the second step was to screen the loci that were not “F” type in the five parents from the results of the first step; the third step was to screen the loci with “high” contribution. Finally, the candidate loci associated with hemorrhagic disease were identified.

Functional verification of susceptible genes related to hemorrhagic disease

The genome annotation information of candidate loci of rare minnow was used to obtain the genes corresponding to these sites. Then, the cDNA sequences of these genes were compared with the annotated information of the grass carp genome [62], and homologous genes in grass carp were selected. In order to preliminarily and quickly verify the function of these candidate loci, we carried out relevant studies at the cell level of grass carp using knockdown technology. For each homologous grass carp gene, siRNA was designed and synthesized by RiboBio Co. Guangzhou. qPCR primers for homologous grass carp genes were designed to confirm the knockdown effect of the siRNA.

A monolayer of GCO cells was subcultured in 24-well plates. When the cells reached 80% confluence at the bottom of the well, siRNA was transfected into the cells using FishTransH (Meisent Co. Wuhan). The dosage of siRNA (concentrated at 20 μmol/L) was 40 pmol per well. Cells were collected at 0 h and 48 h post-transfection, and total RNA was extracted using TRIzol (Life Technologies). RT-qPCR was used to detect the expression of 23 grass carp genes at 48 h relative to 0 h post-transfection. siRNAs with inhibitory effects were selected for the subsequent experiments.

The GCO cells were subcultured in 24-well plates. When the cells reached approximately 80%

confluence at the bottom of the well, the selected siRNA was transfected into the cells using FishTransH. siRNA-NC (RiboBio Co. Guangzhou) was used as a negative control in each group, and the dosage of siRNA was 40 pmol per well. At 16 h post-transfection, the medium was removed and the cells were infected with GCRV at a MOI of 5. The cells were collected 32 h after infection. Total RNA was extracted, and RT-qPCR was performed to detect the relative changes of GCRV RNA relative to the negative control. GCO cells transfected and infected in the same way were removed to −70 °C, and frozen and thawed two times for collecting viral samples. Then, CIK cells were seeded into 96-well plates, 5000 cells per well. After 24 h, the cells per well were infected with 100 μl viral samples of 10-fold serial dilutions in culture medium and incubated for 3 days. CPE was then observed under the microscope, and the titer was determined using the Reed-Muench formula [63] and expressed as $TCID_{50}$/ml.

Data Availability

Raw sequences for genome assembly including Illumina, PacBio and Hi-C reads have been deposited in NCBI under accession number PRJNA732062. Sequencing data for screening of candidate indels associated with hemorrhagic disease also has been deposited in the NCBI under accession numbers PRJNA732511 and PRJNA613868. The genome and annotation files of rare minnow are available from Dryad (https://datadryad.org/stash/share/MnVhBHY9w4PKd7VshDbgrgdZGKPW_6NWYMGVPhtVFr4) with a DOI (doi:10.5061/dryad.jh9w0vtb0).

Additional Files

Additional File 1: Table. Statistics of repeat elements.

Additional File 2: Table. Gene annotation statistics for four Cyprinidae fish species.

Additional File 3: Table. Twenty-three loci associated with hemorrhagic diseases.

Additional File 4: Table. Distance from 23 loci to target sites.

Additional File 5: Table. Twenty genes associated with hemorrhagic diseases.

Additional File 6: Table. Twenty-three homologous genes in grass carp.

Additional File 7: Table. siRNAs and specific primer sequences for the 23 grass carp genes.

Additional File 8: Figure. Pipeline of random genome editing.

Abbreviations

CCTCC: China Center for Type Culture Collection; CDS: coding sequence; CIK: *Ctenopharyngodon idellus* kidney; CPE: cytopathic effect; CRISPR: clustered regularly interspersed short palindromic repeats; dpi: days post-infection; ENU: ethyl nitrosouria; GCO: grass carp ovary; GCRV: grass carp reovirus; ML: maximum likelihood; NC: negative control; NCBI: National Center for Biotechnology Information; NGS: next-generation sequencing; PacBio: Pacific Biosciences; sgRNA: small guide RNA; SNP: single nucleotide polymorphisms; SNR: signal-to-noise ratio; SRA: sequence read archive; TGS: third-generation sequencing.

Competing Interests

The authors declare that they have no competing interests.

Funding

This work was supported by the National Natural Science Foundation of China (31972788) and the State of Key Laboratory of Freshwater Ecology and Biotechnology (2019FBZ05, 2021FB11).

Authors' Contributions

R.H. and Y.W. conceived and designed the experiments. L.F.L., R.H., M.O. and Y.L. performed the experiments. M.S., C.Y., W.Z. and X.X. analyzed the genome data. R.H., M.S. and L.F.L. drafted and revised the manuscript. Y.W., L.J.L. and Z.Z. provided advice on manuscript writing. All authors reviewed the manuscript.

References

1. Liao T, Xu Y, Zhong XP, et al. Comparative vitellogenic responses in zebrafish (*Brachydanio rerio*) and rare minnow (*Gobiocypris rarus*) exposed to 17α-ethinylestradiol. Acta Hydrobiologica Sinica 2005;**29**(5):513–7.
2. Jiang FQ. Methods of acute and subchronic toxicity test using *Gobiocypris rarus* and their application

in toxicity study of sediment in Donghu lake. Beijing: Graduate School of Chinese Academy of Sciences 2006.

3. Zha JM, Sun LW, Spear PA, et al. Comparison of ethinylestradiol and nonylphenol effects on reproduction of Chinese rare minnows (*Gobiocypris rarus*). Ecotoxicology and Environmental Safety 2008;**71**(2):390–9.
4. Wang J, Cao W. *Gobiocypris rarus* as a chinese native model organism: history and current situation. Asian Journal of Ecotoxicology 2017;**12**:20–33.
5. Wang T, Liu P, Chen H, et al. Preliminary study on the susceptible of *Gobiocypris rarus* to hemorrhagic virus of grass carp (GCHV). Acta Hydrobiologica Sinica 1994;**2**:144–9.
6. Zhang L, Luo Q, Fang Q, et al. An improved RT-PCR assay for rapid and sensitive detection of grass carp reovirus. J Virol Methods 2010;**169**(1):28–33.
7. Sega GA. A review of the genetic effects of ethyl methanesulfonate. Mutat Res 1984;**134**(2-3):113-42.
8. McCallum CM, Comai L, Greene EA, et al. Targeted screening for induced mutations. Nat Biotechnol 2000;**18**(4):455–7.
9. Till BJ, Reynolds SH, Greene EA, et al. Large-scale discovery of induced point mutations with high-throughput TILLING. Genome Res 2003;**13**(3):524–30.
10. Singh M, Lewis PE, Hardeman K, et al. Activator mutagenesis of the pink scutellum1/viviparous7 locus of maize. Plant Cell 2003;**15**(4):874–84.
11. Bai L, Singh M, Pitt L, et al. Generating novel allelic variation through Activator insertional mutagenesis in maize. Genetics 2007;**175**(3):981–92.
12. Ran FA, Hsu PD, Wright J, et al. Genome engineering using the CRISPR-Cas9 system. Nat Protoc 2013;**8**(11):2281–308.
13. Mehravar M, Shirazi A, Nazari M, et al. Mosaicism in CRISPR/Cas9-mediated genome editing. Dev Biol 2019;**445**(2):156–62.
14. Zhou Y, Zhu S, Cai C, et al. High-throughput screening of a CRISPR/Cas9 library for functional genomics in human cells. Nature 2014;**509**(7501):487–91.
15. Kim HS, Lee K, Bae S, et al. CRISPR/Cas9-mediated gene-knockout screens and target identification via whole genome sequencing uncover host genes required for picornavirus Infection. J Biol Chem

2017;**292**(25):10664–71.

16. Lu Y, Ye X, Guo R, et al. Genome-wide targeted mutagenesis in rice using the CRISPR/Cas9 system. Mol Plant 2017;**10**(9):1242–5.
17. Meng X, Yu H, Zhang Y, et al. Construction of a genome-wide mutant library in rice using CRISPR/Cas9. Mol Plant 2017;**10**(9):1238–41.
18. Yang C, Bolotin E, Jiang T, et al. Prevalence of the initiator over the TATA box in human and yeast genes and identification of DNA motifs enriched in human TATA-less core promoters. Gene 2007;**389**(1):52–65.
19. Zhang XH, Qi YX. Analysis on TATA-box, GC-box and CAAT-box in eukaryotic promoters. Journal of Anhui Agri Sci 2008;**36**(4):1380–95.
20. Ren T, Ren H, Du H, et al. Construction of EMS mutant library and screening of total flavonoid content mutants of apocynum venetum. Journal f of Plant Genetic Resources 2020;**21**(3):655–62.
21. Shan C, Shu Q, Wu D. Preliminary study on adding to leaf color marker for rice cytoplasmic male sterile (cms) line long-te-fu A by mutation technology. Journal of Zhejiang University (Agric.& Life Sci.) 1999;**25**:569–72.
22. Xin Z, Wang ML, Barkley NA, et al. Applying genotyping (TILLING) and phenotyping analyses to elucidate gene function in a chemically induced sorghum mutant population. BMC Plant Biol 2008;**8**(1):103.
23. Julio E, Laporte F, Reis S, et al. Reducing the content of nornicotine in tobacco via targeted mutation breeding. Mol Breeding 2008;**21**(3):369–81.
24. Wilson K, Long D, Swinburne J, et al. A dissociation insertion causes a semidominant mutation that increases expression of *TINY*, an arabidopsis gene related to *APETALA2*. Plant Cell 1996;**8**(4):659–71.
25. Marsch-Martinez N, Greco R, Van Arkel G, et al. Activation tagging using the *En-I* maize transposon system in Arabidopsis. Plant Physiol 2002;**129**(4):1544–56.
26. De Stasio EA, Dorman S. Optimization of ENU mutagenesis of *Caenorhabditis elegans*. Mutat Res 2001;**495**(1-2):81–8.
27. Epstein HF, Shakes DC. *Caenorhabditis elegans*: modern biological analysis of an organism. 1st ed. Academic Press; 1995.

28. Driever W, Solnica-Krezel L, Schier AF, et al. A genetic screen for mutations affecting embryogenesis in zebrafish. Development 1997;**123**:37–46.

29. Geisler R, Rauch GJ, Geiger-Rudolph S, et al. Large-scale mapping of mutations affecting zebrafish development. BMC Genomics 2007;**8**(1):11.

30. Hrabe de Angelis MH, Flaswinkel H, Fuchs H, et al. Genome-wide, large-scale production of mutant mice by ENU mutagenesis. Nat Genet 2000;**25**(4):444–7.

31. Concepcion D, Seburn KL, Wen G, et al. Mutation rate and predicted phenotypic target sizes in ethylnitrosourea-treated mice. Genetics 2004;**168**(2):953–9.

32. Jiang XY, Sun CF, Zhang QG, et al. ENU-induced mutagenesis in grass carp (*Ctenopharyngodon idellus*) by treating mature sperm. PLoS One 2011;**6**(10):e26475.

33. Hai T, Cao C, Shang H, et al. Pilot study of large-scale production of mutant pigs by ENU mutagenesis. Elife 2017;**6**:e26248.

34. Koren S, Walenz B P, Berlin K, et al. Canu: scalable and accurate long-read assembly via adaptive k-mer weighting and repeat separation. Genome Res 2017;**27**(5):722–36.

35. Ruan J, Li H. Fast and accurate long-read assembly with wtdbg2. Nat Methods 2020;**17**(6):155–8.

36. Walker BJ, Abeel T, Shea T, et al. Pilon: an integrated tool for comprehensive microbial variant detection and genome assembly improvement. PloS One 2014;**9**(11):e112963.

37. Servant N, Varoquaux N, Lajoie BR, et al. HiC-Pro: an optimized and flexible pipeline for Hi-C data processing. Genome Biol 2015;**16**:259.

38. Burton JN, Adey A, Patwardhan RP, et al. Chromosome-scale scaffolding of de novo genome assemblies based on chromatin interactions. Nat Biotechnol 2013;**31**(12):1119–25.

39. Simão FA, Waterhouse RM, Ioannidis P, et al. BUSCO: assessing genome assembly and annotation completeness with single-copy orthologs. Bioinformatics 2015;**31**(19):3210–2.

40. Chen N. Using RepeatMasker to identify repetitive elements in genomic sequences. Curr Protoc Bioinformatics 2004;**4**(4):10.

41. Bao WD, Adey A, Patwardhan RP. Repbase Update, a database of repetitive elements in eukaryotic genomes. Mob DNA 2015;**6**(1):11.

42. Stanke M, Diekhans M, Baertsch R, et al. Using native and syntenically mapped cDNA alignments to

improve de novo gene finding. Bioinformatics 2008;**24**(5):637–44.

43. Allen JE, Majoros WH, Pertea M, et al. JIGSAW, GeneZilla, and GlimmerHMM: puzzling out the features of human genes in the ENCODE regions. Genome Biol 2006;**7**(1):1–13.
44. Blanco E, Abril JF. Computational gene annotation in new genome assemblies using GeneID. Methods Mol Biol 2009;**537**(537):243-61.
45. Korf I. Gene finding in novel genomes. BMC bioinformatics 2004;**5**(1):59.
46. Keilwagen J, Wenk M, Erickson JL, et al. Using intron position conservation for homology-based gene prediction. Nucleic Acids Res 2016;**44**(9):e89.
47. Keilwagen J, Hartung F, Paulini M, et al. Combining RNA-seq data and homology-based gene prediction for plants, animals and fungi. BMC Bioinformatics 2018;**19**(1):189.
48. Kim D, Langmead B, Salzberg SL. HISAT: a fast spliced aligner with low memory requirements. Nat Methods 2015;**12**(4):357–60.
49. Pertea M, et al. StringTie enables improved reconstruction of a transcriptome from RNA-seq reads. Nat Biotechnol. 2015;**33**(3):290–5.
50. Grabherr MG, Haas BJ, Yassour M, et al. Full-length transcriptome assembly from RNA-seq data without a reference genome. Nat Biotechnol 2011;**29**(7):644–52.
51. Campbell MA, Haas BJ, Hamilton JP, et al. Comprehensive analysis of alternative splicing in rice and comparative analyses with Arabidopsis. BMC genomics 2006;**7**(1):327.
52. Haas BJ, Salzberg SL, Zhu W, et al. Automated eukaryotic gene structure annotation using EVidenceModeler and the program to sssemble spliced alignments. Genome Biol 2008,**9**(1):R7.
53. Emms D M, Kelly S. OrthoFinder: phylogenetic orthology inference for comparative genomics. Genome biol 2019;**20**(1):238.
54. Mi H, Muruganujan A, Ebert D, et al. PANTHER version 14: more genomes, a new PANTHER GO-slim and improvements in enrichment analysis tools. Nucleic Acids Res 2019;**47**(D1):D419–26.
55. Katoh K, Asimenos G, Toh H. Multiple alignment of DNA sequences with MAFFT. Methods Mol Biol 2009;**537**:39–64.
56. Kozlov AM, Darriba D, Flouri T, et al. RAxML-NG: A fast, scalable, and user-friendly tool for maximum likelihood phylogenetic inference. Bioinformatics 2019;**35**(21):4453–5.

57. Han MV, Thomas GW, Lugo-Martinez J, et al. Estimating gene gain and loss rates in the presence of error in genome assembly and annotation using CAFE 3. Mol Biol Evol 2013;**30**(8):1987–97.
58. Huang X, Jiang Y, Zhang W, et al. Construction of a high-density genetic map and mapping of growth related QTLs in the grass carp (*Ctenopharyngodon idellus*). BMC genomics 2020;**21**(1):313.
59. Tang H, Krishnakumar V, Li J. jcvi: JCVI utility libraries. 2015.
60. Langmead B, Salzberg SL. Fast gapped-read alignment with Bowtie 2. Nat Methods 2012;**9**(4):357–9.
61. Mckenna A, Hanna M, Banks E, et al. The Genome Analysis Toolkit: A MapReduce framework for analyzing next-generation DNA sequencing data. Genome Res 2010;**20**(9):1297–303.
62. Wang Y, Lu Y, Zhang Y, et al. The draft genome of the grass carp (*Ctenopharyngodon idellus*) provides genomic insights into its evolution and vegetarian diet adaptation. Nat Genet 2015;**47**(6):625–31.
63. Lindenbach BD. Measuring HCV infectivity produced in cell culture and in vivo. Methods Mol Biol 2009;**510**:329–36.

Table and figure captions

Table 1: Summary statistics of the rare minnow reference genome assembly

Figure 1: Evolutionary analysis of the genome of rare minnow. a. Rare minnow genome contact matrix using Hi-C data. The color bar illuminates the logarithm of the contact density from red (high) to white (low) in the plot. Note that only sequences anchored on chromosomes are shown in the plot. b. A phylogenetic tree was constructed from 12 species, including the four Cyprinidae species. The time of divergence and the expansion and contraction of gene families of the four Cyprinidae species are described with a maximum-likelihood tree. The number of expansion events is indicated in red, and contraction events are indicated in green. c. A comparative analysis of the rare minnow and grass carp genomes was performed. There was a high collinearity between the two species. Rare minnow LG1 and LG21 corresponded to grass carp LG13. The LG number and supercoiling number of grass carp were obtained from the study of Huang et al. [58].

Figure 2: Establishment of an anti-hemorrhagic model of rare minnow. a. Survival rates of the P0 mutant

group and control group after GCRV infection. The P0 mutant group and control group were similarly infected with GCRV via the high-salt invasion method. b. Clinical symptoms of the mutant and control groups after GCRV infection. There was no difference in the clinical phenotype between the mutant group and the control group that died after GCRV infection. The body surface of the dead individuals in both the mutant and control groups was red, showing obvious symptoms of hemorrhagic disease. c. Survival rates of the F1–F3 and control groups after GCRV infection. The high-salt invasion method was used. d–f. Daily cumulative mortality for the F1, F2, F3, and control groups after GCRV infection. The number of dead fish in all groups was recorded every day. The number of the daily cumulative deaths in each group relative to the total number of individuals in each group is the daily cumulative mortality. Different colors were used to represent different families. The abscissa represents the days post-infection and the ordinate represents the cumulative mortality.

Figure 3: Screening of candidate indels related to hemorrhagic disease. “T” denotes new genotypes compared with those in the control group, “N” denotes no results of genotyping, and “F” indicates that the genotypes also appear in the control group. The outer ring shows the statistics of the genotyping results of three F2 families; “TTN” indicates that the first and the second families have new genotypes and the third family has no genotyping results; “TFT” indicates that the first and third families have new genotypes, and the second family's genotypes also appear in the control group, and so on. The inner ring indicated the genotyping results of 147,679 loci (the sum of TTN, NTT, TNT, TNN, NTN, NNT, and TTT type in the outer ring) in five parents (P1–P5). “F0” means that there are either new genotypes or no genotyping results among the five parents compared with that in the control group. “F1” indicates that one of the five parents has the same genotypes as the control group, while the other four parents have either new genotype or no genotyping results, and so on.

Figure 4: Effects of siRNAs on screened target genes and GCRV. a. The GCO cells were cultured in 24-well plates. Each siRNA for grass carp genes was transfected into the cells. The cellular total RNA was extracted at 0 h and 48 h post-transfection. q-PCR was used to detect the relative expression of 23 grass cap genes using beta actin as the internal reference gene and $2^{-\Delta\Delta Ct}$ method. The ratios of 48 h/0 h of each group were then calculated. b. The selected nine siRNAs were transfected into the GCO cells. The siRNA negative control (NC) was used in each group. At 16 h post-transfection, the medium was removed, and the

cells were infected with GCRV with MOI = 5. The cells were collected at 32 h post-infection. Using beta actin as the internal reference gene, the relative expression of GCRV RNA relative to NC was detected by the $2^{-\Delta\Delta Ct}$ method. c. CIK cells were seeded into 96-well plates. Then, the cells were infected with viral samples (from GCO cells transfected and infected as in b) for 3 days. CPE was then observed under the microscope, and the titer was determined using the Reed-Muench formula. Data represent results of three independent experiments, and error bars indicate mean ± SD. Statistical analyses were performed using multiple t-tests (n = 3), and asterisk indicates $P < 0.05$.

Figure 5: Design of the upstream primers for the 26 target sites. a. Eleven forward sgRNA primer sequences designed with TATAWAW as the target. b. Fifteen forward sgRNA primer sequences designed with ATG as the target, where M=A/C, R=A/G, W=A/T, Y=C/T, N=A/T/C/G; Red and blue sequences represent the whole target sequence region, which starts with GGR to improve transcription efficiency. blue sequences represent the conservative sequences of the promoter region (a) and the start codon sequences (b).

Click here to access/download;Table;Table 1.docx

TABLE 1 Summary statistics of the rare minnow reference genome assembly

Assembly	Contig number	Contig length (bp)	Scaffold number	Scaffold length (bp)
N50	48	5,468,461	12	36,585,240
N90	203	896,652	23	28,204,685
Max	1	25,522,336	1	53,027,249
Total	858	960,267,999	566	959,102,419
Anchored to chromosomes	694	956,050,416 (99.56%)	345	924,697,551 (96.41%)

Figure 1

Click here to access/download;Figure;Fig. 1.tif

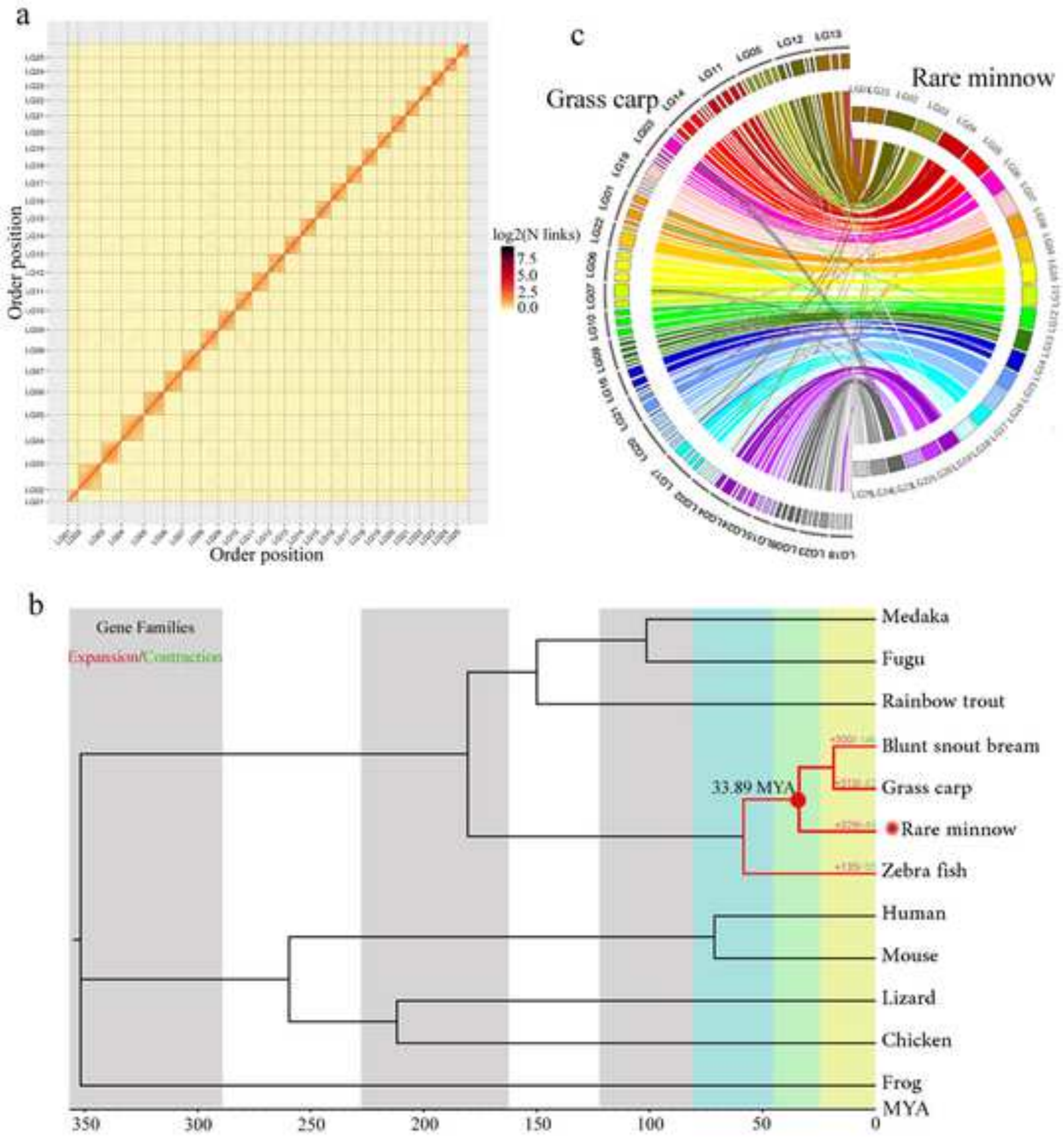

Figure Click here to access/download;Figure;Fig. 2.tif

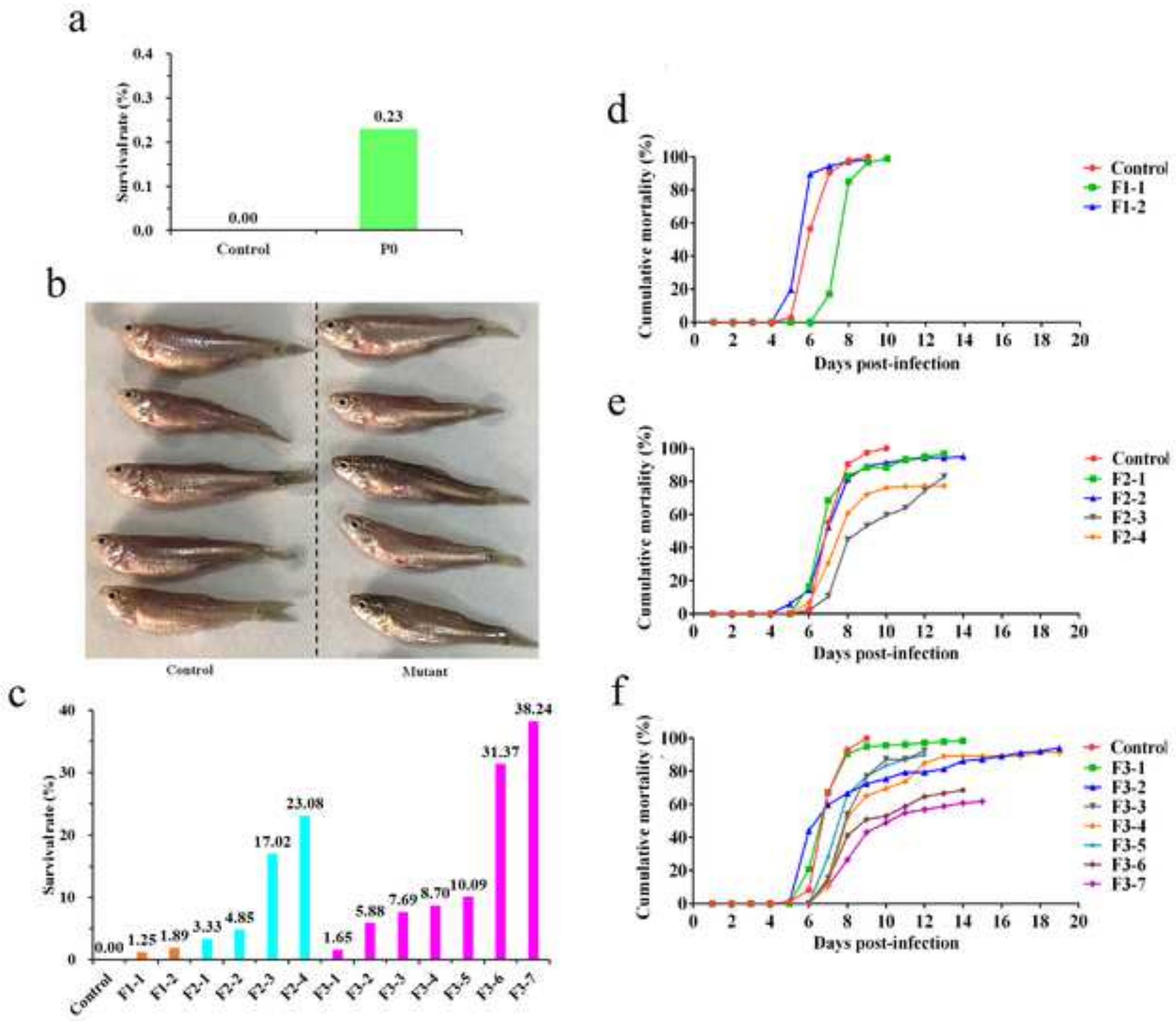

Figure

Click here to access/download;Figure;Fig. 3.tif

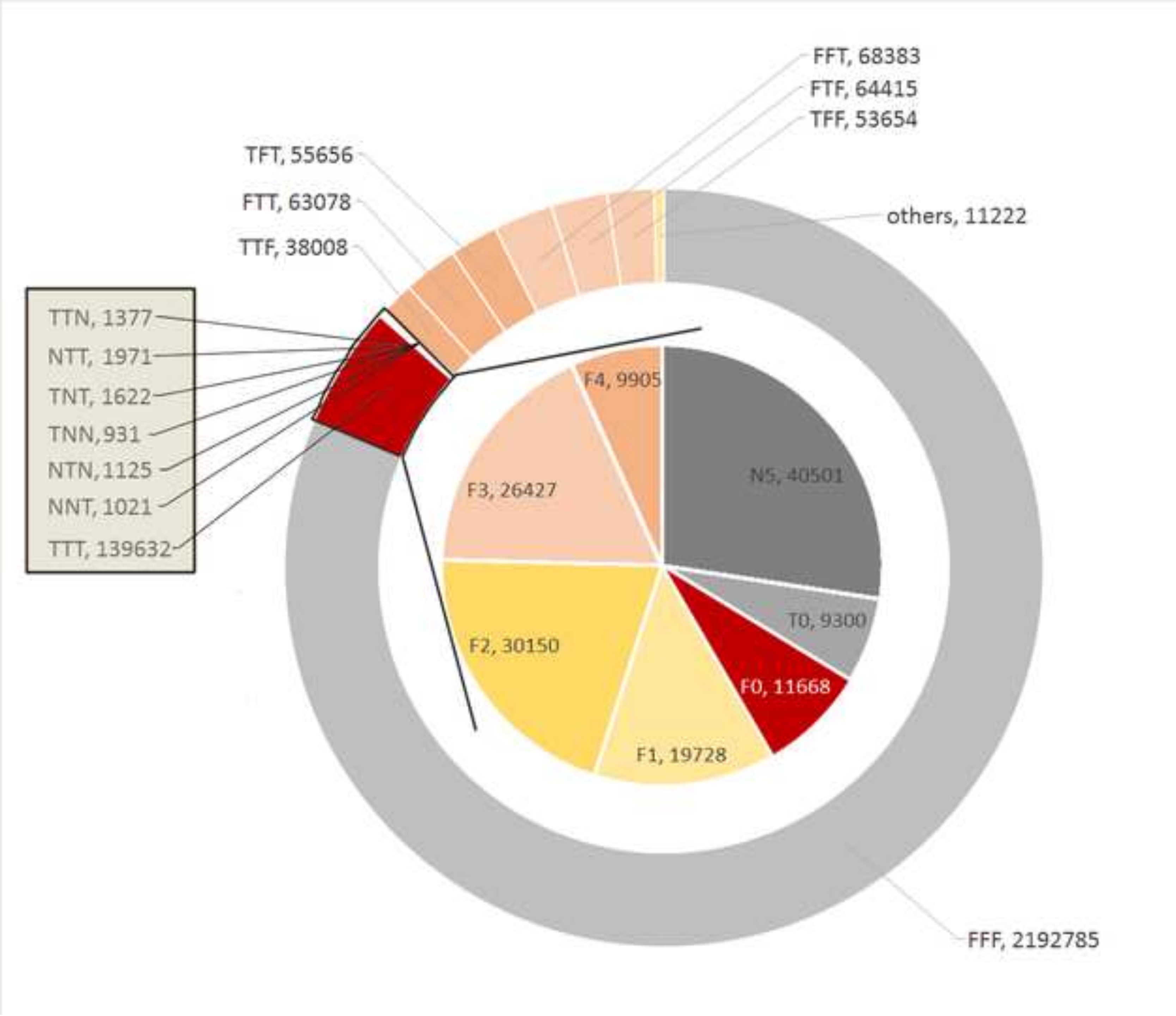

Figure	Click here to access/download;Figure;Fig. 4.tif

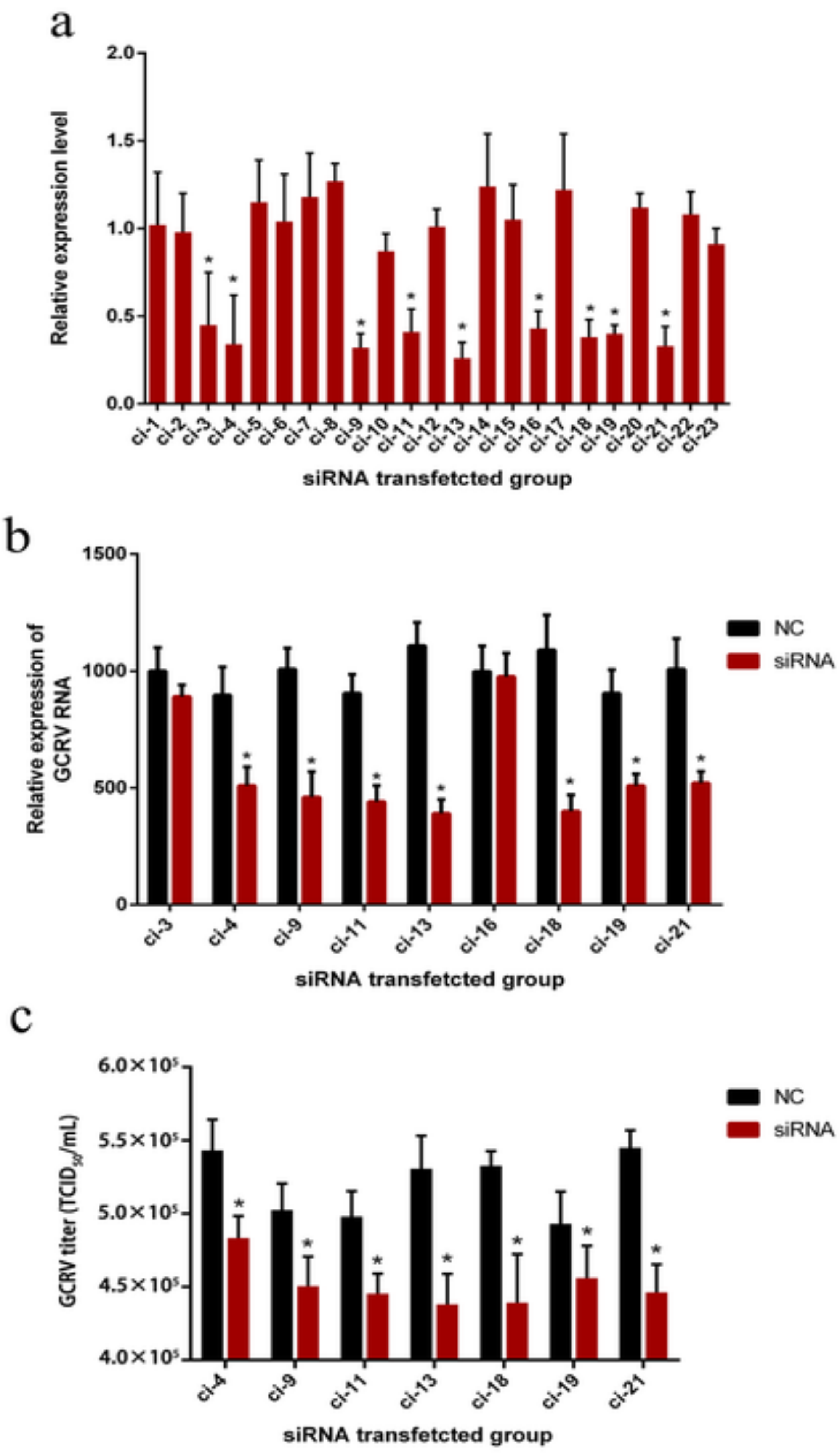

Figure
Click here to access/download;Figure;Fig. 5.tif

a

TATA0: TGTAATACGACTCACTATAGGRTATAWAWNNNNNNNNNNGTTTTAGAGCTAGAAAT
TATA1: TGTAATACGACTCACTATAGGRNTATAWAWNNNNNNNNNGTTTTAGAGCTAGAAAT
TATA2: TGTAATACGACTCACTATAGGRNNTATAWAWNNNNNNNNGTTTTAGAGCTAGAAAT
TATA3: TGTAATACGACTCACTATAGGRNNNTATAWAWNNNNNNNGTTTTAGAGCTAGAAAT
TATA4: TGTAATACGACTCACTATAGGRNNNNTATAWAWNNNNNNGTTTTAGAGCTAGAAAT
TATA5: TGTAATACGACTCACTATAGGRNNNNNTATAWAWNNNNNGTTTTAGAGCTAGAAAT
TATA6: TGTAATACGACTCACTATAGGRNNNNNNTATAWAWNNNNGTTTTAGAGCTAGAAAT
TATA7: TGTAATACGACTCACTATAGGRNNNNNNNTATAWAWNNNGTTTTAGAGCTAGAAAT
TATA8: TGTAATACGACTCACTATAGGRNNNNNNNNTATAWAWNNGTTTTAGAGCTAGAAAT
TATA9: TGTAATACGACTCACTATAGGRNNNNNNNNNTATAWAWNGTTTTAGAGCTAGAAAT
TATA10: TGTAATACGACTCACTATAGGRNNNNNNNNNNTATAWAWGTTTTAGAGCTAGAAAT

b

ATG0: TGTAATACGACTCACTATAGGRATGNNNNNNNNNNNNNNGTTTTAGAGCTAGAAAT
ATG1: TGTAATACGACTCACTATAGGRNATGNNNNNNNNNNNNNGTTTTAGAGCTAGAAAT
ATG2: TGTAATACGACTCACTATAGGRNNATGNNNNNNNNNNNNGTTTTAGAGCTAGAAAT
ATG3: TGTAATACGACTCACTATAGGRNNNATGNNNNNNNNNNNGTTTTAGAGCTAGAAAT
ATG4: TGTAATACGACTCACTATAGGRNNNNATGNNNNNNNNNNGTTTTAGAGCTAGAAAT
ATG5: TGTAATACGACTCACTATAGGRNNNNNATGNNNNNNNNNGTTTTAGAGCTAGAAAT
ATG6: TGTAATACGACTCACTATAGGRNNNNNNATGNNNNNNNNGTTTTAGAGCTAGAAAT
ATG7: TGTAATACGACTCACTATAGGRNNNNNNNATGNNNNNNNGTTTTAGAGCTAGAAAT
ATG8: TGTAATACGACTCACTATAGGRNNNNNNNNATGNNNNNNGTTTTAGAGCTAGAAAT
ATG9: TGTAATACGACTCACTATAGGRNNNNNNNNNATGNNNNNGTTTTAGAGCTAGAAAT
ATG10: TGTAATACGACTCACTATAGGRNNNNNNNNNNATGNNNNGTTTTAGAGCTAGAAAT
ATG11: TGTAATACGACTCACTATAGGRNNNNNNNNNNNATGNNNGTTTTAGAGCTAGAAAT
ATG12: TGTAATACGACTCACTATAGGRNNNNNNNNNNNNATGNNGTTTTAGAGCTAGAAAT
ATG13: TGTAATACGACTCACTATAGGRNNNNNNNNNNNNNATGNGTTTTAGAGCTAGAAAT
ATG14: TGTAATACGACTCACTATAGGRNNNNNNNNNNNNNNATGGTTTTAGAGCTAGAAAT

Click here to access/download
Supplementary Material
Additional File 1.xlsx

Click here to access/download
Supplementary Material
Additional File 2.xlsx

Click here to access/download
Supplementary Material
Additional File 3.xlsx

Click here to access/download
Supplementary Material
Additional File 4.xlsx

Click here to access/download
Supplementary Material
Additional File 5.xlsx

Click here to access/download
Supplementary Material
Additional File 6.xlsx

Click here to access/download
Supplementary Material
Additional File 7.xlsx

Click here to access/download
Supplementary Material
Additional File 8.tif
