# Supplementary figures and images for: De novo screening of disease-resistant genes from the chromosome-level genome of rare minnow using CRISPR-cas9 random mutation
Source: Gigascience. 2021 Nov 19;10(11):giab075. doi: 10.1093/gigascience/giab075 (PMC8782236; doi:10.1093/gigascience/giab075)

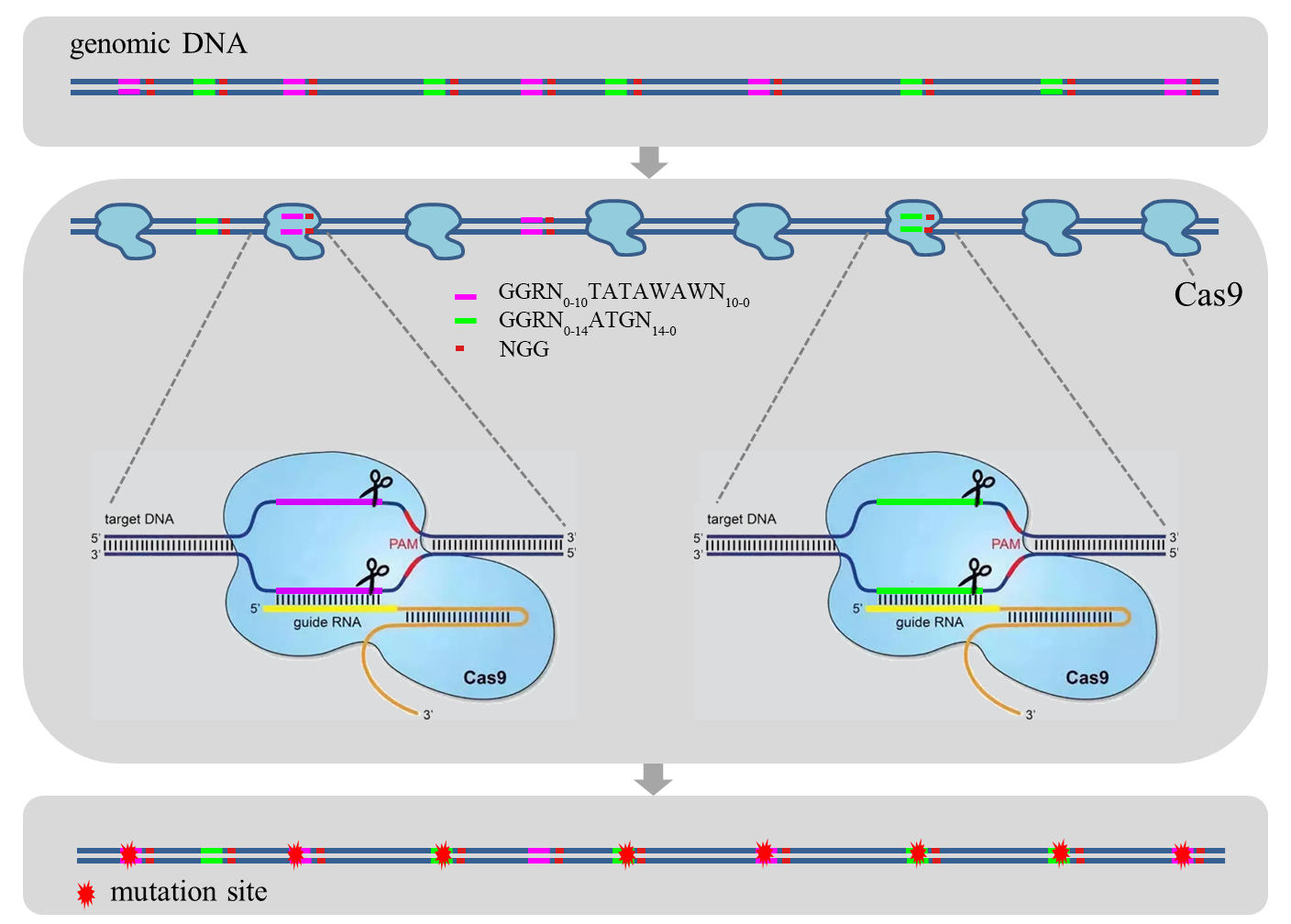

Supplement: giab075_Supplemental_Files [file giab075_supplemental_files.zip › Additional File 8.tif]
