# Supplementary material for: De novo screening of disease-resistant genes from the chromosome-level genome of rare minnow using CRISPR-cas9 random mutation
Source: Gigascience. 2021 Nov 19;10(11):giab075. doi: 10.1093/gigascience/giab075 (PMC8782236; doi:10.1093/gigascience/giab075)
Supplement: giab075_Response_to_Reviewer_Comments_Original_Submission [file giab075_response_to_reviewer_comments_original_submission.pdf]

Author's Response To Reviewer Comments

Close

GIGA-D-21-00147
De novo screening of disease-resistant genes at the chromosome-level genome of rare minnow using CRISPR/cas9 random mutation
Lifei Luo; Mijuan Shi; Cheng Yang; Mi Ou; Wanting Zhang; Lanjie Liao; Yongming Li; Xiaoqin Xia; Zuoyan Zhu; Rong Huang; Yaping Wang
GigaScience

Dear Mr. 汪,
Your manuscript "De novo screening of disease-resistant genes at the chromosome-level genome of rare minnow using CRISPR/cas9 random mutation" (GIGA-D-21-00147) has been assessed by our reviewers (sorry for the delay - we needed three reviewers in this case, to cover both aspects of your paper, the CRSIPR technique and whole genome assembly).
Although the paper is of interest, we are unable to consider it for publication in its current form. The reviewers have raised a number of points which we believe would improve the manuscript and may allow a revised version to be published in GigaScience.
Their reports are below.
If you are able to fully address these points, we would encourage you to submit a revised manuscript to GigaScience. Once you have made the necessary corrections, please submit online at:
https://www.editorialmanager.com/giga/
Please make sure to include BUSCO analysis, using the latest version of the software. BUSCO output files should be stored on our server, for release via GigaDB in case of acceptance.
If you have forgotten your username or password please use the "Send Login Details" link to get your login information. For security reasons, your password will be reset.
Please include a point-by-point within the 'Response to Reviewers' box in the submission system. Please ensure you describe additional experiments that were carried out and include a detailed rebuttal of any criticisms or requested revisions that you disagreed with. Please also ensure that your revised manuscript conforms to the journal style, which can be found in the Instructions for Authors on the journal homepage. If the data and code has been modified in the revision process please be sure to update the public versions of this too.
The due date for submitting the revised version of your article is 01 Nov 2021.
I look forward to receiving your revised manuscript soon.
Best wishes,
Hans Zauner
GigaScience
www.gigasciencejournal.com
Answer: Thank you for giving us a chance to resubmit this manuscript, and also thank the reviewers for giving us constructive suggestions, which have helped us both in English and in depth to improve the quality of the paper. We have performed genome assessment using BUSCO v5.2.2 and uploaded the output files to GigaDB via FTP. At the same time, the relevant results were added to the revised manuscript and marked in blue font.

Reviewer reports:
Reviewer #1: In this study, "De novo screening of disease-resistant genes at the chromosome-level genome of rare minnow using CRISPR/cas9 random mutation" Luo et al. reported a chromosome-level genome of wild-type rare minnow and a new approach of mutagenesis using universal guide RNAs for CRISPR-Cas9. Compared to genome-wide CRISPR knockout (GeCKO) screening, this approach does not require species-specific guide RNA libraries as the universal gRNA are designed to target all possible promoters or start codon regions. It demonstrated that candidate genes for disease resistance can be found using this approach. While this study presents a novel and useful approach of de novo screening of disease-resistant genes, I found some aspects that could be improved.

In page 7, in the 3rd paragraph of the Discussion, '..however, the disadvantage is that it requires sufficient candidate gene sequences.', it is true but the method in the paper also requires the whole

genome sequence information to analyse the mutations (although it is not required to design gRNAs in your method).
Answer: Thank you for your careful and valuable suggestions. We agree with you. This method needs genome sequence information. We have added this point to the revised manuscript, please see the 4th paragraph of the discussion.

In page 7, in the 4th paragraph of the Discussion, could you clarify what the mutation efficiency means? For example, when you say 0.23% mutation efficiency in rare minnow does it means 0.0023 X 943.44M bp = 2.17 M bp has been mutated in individual fish?
Answer: The "mutation efficiency" here refers to the proportion of individuals with mutation characteristics in a population. This usage is inaccurate and may cause ambiguity, so we have changed it to "mutant frequency". Please see the 4th paragraph of the discussion.

In page 12, in the 3rd paragraph, could you describe the criteria for the four levels of contribution to gene function changes?
Answer: The four levels of contribution to gene function changes were according to the snpEff definition, which are shown in the followd website: https://pcingola.github.io/SnpEff/se_inputoutput/#impact-prediction. We have added this website to the revised manuscript. Please see the 1st paragraph on page 13.

In figure 2 d, e & f, could you consider making X-axis aligned among fig. 2 d, e & f? For example, make all X axis to have 15 days post infection as maximum value or 20 dpi.
Answer: Thank you for your reminder. We have redrawn these diagrams. Please see the Fig. 2d-f in the revised manuscript.

In figure 3, what does arrow means? Could you consider improve the figure 3 and the text in the 2nd paragraph in page 6 ('There were 147,679 (139632 + 1377 + …') for better readability?
Answer: We have modified the figure 3 and the corresponding text description in order to clearly illustrate the filtering process of indels. Please see the figure 3 and the text in the 2nd paragraph on page 6 of the revised manuscript.

The viral loads were around 2 fold reduced in the knock-down (KD) grass carp cells for 7 target genes. However, 2 fold reduction in viral copy numbers is considered to be a subtle change and more importantly the viral load cannot confirm whether the viral infectivity was reduced. Analysing the infectivity of the virus produced from the KD cells by TCID50 or plaque assay together with the viral load is recommended.
Answer: Thank you for your constructive suggestions. We have supplemented experiments to analyze the infectivity of the virus produced from the KD cells by TCID50. The results showed that inhibition of the expression of these seven genes did affect the titer of GCRV. Please see the Fig. 4c in the revised manuscript.

Regarding the viral load experiment, have you checked the viral load in the resistant and susceptible minnow in vivo? If so, was there a difference in viral load between the resistant and susceptible fish?
Answer: Since we have not yet obtained 100% resistant families (All individuals can survive after infection), this means that we cannot know which individuals are resistant to disease before infection. After infection, the surviving resistant individuals are likely to have a certain immune memory or antibody level. If the surviving resistant individuals and wild individuals are infected with virus at the same time again, the real differences in viral load between the resistant and susceptible fish may not be obtained due to the different immune backgrounds of the host. I don't know whether our consideration is reasonable. Thank you.

I'm wondering why did you knock down the targets in grass carp cells instead of knockout? Knockout would be more suitable to validate the function of your candidate genes.
Answer: Thank you for your valuable suggestions. Because knock down can verify the gene function faster and more comprehensively, and the knock down results have proved that the genes we screened are effective. Knocking out of 23 genes is a time-consuming process. In following study, we will use knockout technology to further verify the function of candidate genes.

Could you delineate the limitation of this approach in the discussion? For examples, for species with long generation time or screening for quantitative traits, this may not be feasible and etc.
Answer: We have added relevant comments in the 4th paragraph of the discussion in the revised manuscript.

gRNAs were designed to target all protein coding genes but as the gRNA quantity for each target region is extremely low, so I think the mutations most likely happen to 'easy to target loci' which could leave numerous protein coding genes unmutated. Did you check how many genes were mutated in the founder fish before the viral challenge (to check the genome-wide mutation coverage of this 26 gRNA system)?
Answer: Thank you and we agree with you. Although this mutation method is aimed at a large number of protein coding genes, in fact, due to the limited number of gRNA, we cannot guarantee that all genes could be mutated. Because the mutation sites of different founder fish were different, and there was no suitable control population for comparison, we did not detect the mutation sites of the founder fish. However, we found that the number of possible mutation sites in disease-resistant fish was 11,668, covered by 2,441 genes, accounting for 9.09% (2,441/26,861) of the total annotated genes.

Typos or minor changes:
P6, 2nd paragraph, '..(inner ring of Fi. 3).' to be '..(inner ring of Fig. 3).'
Answer: We have modified it according to your suggestion. Please see the 2nd paragraph on page 6 in the revised manuscript.

P12, 3rd paragraph, '…without a typing result..' to be '…without a genotyping result..'.
Answer: We have modified it according to your suggestion. Please see the 1st paragraph on page 13 in the revised manuscript.

P13, 2nd paragraph, 'Genes with inhibitory effects…' to be 'siRNAs with inhibitory effects…'.
Answer: We have modified it according to your suggestion. Please see the 3rd paragraph on page 13 in the revised manuscript.

P3, 3rd paragraph, please consider use 'CRISPR-Cas9' instead of 'CRISPR/Cas9' as '-' means 'and' while '/' means 'or'.
Answer: We have revised this word in the full text according to your suggestion.

Reviewer #2: This is an interesting experiment using an innovative combination of random CRISPR/Cas9 mutagenesis with GCRV infection as a powerful selection tool to screen for disease relevant genes. Because of such a high degree of degeneracy in the gRNA sequences, it seems unlikely there would be bi-allelic mutations of any given gene in the injected embryos and thus the method would only find genes in which there is a mono-allelic affect. For the same reason, I was initially concerned that the GCRV survival phenotype would be a result from a combination of so many differ individual mutations making it difficult to isolate individual relevant genes. However, it does seem the screening methods described are sufficient to find at least some GCRV relevant genes, and thus this work is definitely useful and very worthwhile to report. I do have a few concerns and questions I hope can be addressed.

I am somewhat skeptical there was value targeting the TATA box considering only ~20% of promoters contain this element. Was it recorded what percentage of the 23 hemorrhagic disease associated loci came from TATAWAW targeting gRNAs in contrast to ATG targeting gRNAs? This information should be included and discussed in the manuscript.
Answer: Thank you for your constructive suggestions. We analyzed the TATAWAW and ATG targets close to 23 hemorrhagic disease-associated loci. It was found that the targets of 22 loci in 23 were ATG, and the targets of one site could not be determined because both TATAWAW and ATG targets were nearby (within 20bp). The proportion of mutations from ATG targets in 23 loci was close to 100%. This result was included and discussed, please see the 2nd paragraph on page 6 and the 4th paragraph on page 7 in the revised manuscript.

In the "Functional verification of susceptible genes related to hemorrhagic disease" section of the results, the 23 candidate genes were narrowed down to 9. The wording was not clear, you wrote "This indicated that the siRNA of the nine genes had a significant inhibitory effect". I'm assuming you meant, This indicated that nine siRNAs had a significant inhibitory effect".
Answer: We have changed it to "The results indicated that nine siRNAs had a significant inhibitory effect ($p < 0.05$)" according to your suggestion. Please see the "Functional verification of susceptible genes related to hemorrhagic disease" section of the results.

In the methods section, it is not clear why every gRNA sequence was started with an NNR. This should be clarified.
Answer: Thank you for your reminder. Sorry, this is a low-level mistake we made, it should be GGR. Due

to the preference of T7 promoter (used to make gRNAs) for transcription initiation sites, many early studies suggested that transcription should better start with GG, and the third base should better be A or G (Hwang ER et al. 2013; https://www.docin.com/p-915537211.html). In its current implementation, our gRNA-Cas9 system described above can theoretically target the sequence with the form 5'-GGR-N0-10-TATAWAW-N10-0-NGG-3' and 5'-GGR-N0-14-ATG-N14-0-NGG-3'.
References:
1. Hwang WY, Fu Y, Reyon D, et al. Efficient In Vivo Genome Editing Using RNA-Guided Nucleases. Nature Biotechnology, 2013, 31(3):227-229.
2. "Cas9 protocol_v11_130216" in https://www.docin.com/p-915537211.html

As far as functional validation, it seems micro-injection of single gene specific gRNAs targeting the 23 candidate genes from the initial screen would have been most appropriate and should be done in addition to or in place of the siRNA treated GCO cell line validation. What was the rationale behind taking only this latter approach? This rationale should be outlined in the manuscript to better justify the experimental design.
Answer: Thank you for your valuable suggestions. The purpose of gene knockdown in this study is to quickly and effectively verify the screened candidate genes. If knockout is done in rare minnow in vivo, each knockout family needs to be established, it requires at least two generations and more than 8 months. On the other hand, there is no mature CRISPR-cas9 knockout system suitable for fish cell line at present, so the knockout experiment at cell level may not be carried out stably and effectively. In view of the above considerations, this study adopted the experimental design of knockdown of GCO cell line, which can ensure that we can quickly and effectively preliminarily verify the function of candidate genes. We will later use gRNA mediated knockout to deeply analyze the function of candidate genes. We have outlined the rationale in the revised manuscript, please see the 2nd paragraph on page 13.

Reviewer #3: This paper entitled as 'De novo screening of disease-resistant genes at the chromosome-level genome of rare minnow using CRISPR/cas9 random mutation' has shown a novel chromosome-level genome of rare minnow. The authors performed a series of analyses, including genome assembly, annotation, evolution and gene editing. The methods used in this paper are really solid and common.

However, the most significant detect is why this fish is a better gene editing model than zebrafish, which is not clearly described in this study.
Answer: Thank you for your careful review. As a relatively new model fish, rare minnow is not better than zebrafish in every aspect. The reason why the rare minnow was selected as a gene editing model in this study is that it is very sensitive to GCRV virus. The mortality of wild-type rare minnow infected with this virus is 100%, which provides us with an excellent screening method for GCRV resistance, that is, the individuals that can survive after infection may be the individuals with successful mutation. This view was supplementary descripted and explained in the 1st and 5th paragraph of the introduction in the revised manuscript.

On the other hand, the genome assembly has not been evaluated by BUSCO.
Answer: We have performed genome assessment using BUSCO v5.2.2. Please see the "Genome assembly and annotation" section of the Results and the "Genome sequencing and assembly" section of the Methods.

The English language of this paper needs a large of revision.
Answer: We have submitted the revised manuscript to a professional company for language modification and polishing.

In Abstract, the assembled chromosome number should be clarified in this section.
Answer: Thank you for your reminder. We have added the number of assembled chromosomes in this section. Please see the abstract of the revised manuscript.

In Introduction, 'It is worth noting that they are more sensitive to organic pollutants and pathogenic microorganisms than zebrafish (Danio rerio) and medaka (Oryzias latipes).' This sentence should be more clarified why this species is more sensitive. The related reference also should be complemented in this sentence.
Answer: This sentence we wrote was not rigorous, so we have revised it and added references. Please see the 1st paragraph of the introduction in the revised manuscript.

"Traditional physical and chemical mutagenesis methods mainly cause genomic point mutations [4-6], which cannot be distinguished from natural SNP mutations, which leads to considerable difficulties while

performing comparative analysis of the subsequent functional genomes." This sentence should be revised, two 'which' are indeed strange.
Answer: Thank you for your suggestion. We have changed this sentence to “Traditional physical and chemical mutagenesis methods mainly cause genomic point mutations [4-6], which cannot be distinguished from natural SNP mutations, leading to considerable difficulties while performing comparative analysis of the subsequent functional genomes”. Please see the 3rd paragraph of the introduction in the revised manuscript.

'The traditional transposon mutation has' revised to 'The traditional transposon mutations have'.
Answer: We have modified it according to your suggestion. Please see the 3rd paragraph of the introduction in the revised manuscript.

'On one hand, the cost of designing a large number of sgRNAs is high; on the other hand, it is only suitable for the construction of a mutant library of known candidate genes.' should be revised.
Answer: We have changed this sentence to “In this way, a large number of sgRNAs needed be designed at a high cost, and it is only suitable for the construction of a mutant library with known candidate genes” Please see the 4th paragraph of the introduction in the revised manuscript.

'Therefore, the genome of the rare minnow was deemed to be a simple one.' What's real meaning of the 'simple one'?
Answer: "Simple one" means that the repetitive sequence proportion and heterozygosity of the genome are not high, and the genome can be easily sequenced and assembled. For ease of understanding, we have changed this sentence to “The results showed that the genome of rare minnow was a simple genome rather than a complex one”. Please see the “Genome assembly and annotation” section of the Results.

'the N50 was 21,231 bp' should be keep two decimals.
Answer: We have changed it to “the N50 was 21,231.00 bp”. Please see the 2nd paragraph on page 4.

'181,735,272 pairs of unique mapped read pairs were obtained' revise to '181,735,272 pairs of unique mapped reads were obtained'.
Answer: We have modified it according to your suggestion. Please see the 3rd paragraph on page 4.

'a total of 36,387 mRNA encoding proteins were annotated, corresponding to 26,861 genes.' Why 26,861 genes were related to 36387 mRNA? This set contains alternative splicing results?
Answer: Yes, there were 4,957 genes with alternative splicing transcripts. We have supplemented the relevant description, please see the “Genome assembly and annotation” section of the Results.

No BUSCO evaluation result for the genome assembly.
Answer: We have performed genome assessment using BUSCO v5.2.2. Please see the "Genome assembly and annotation" section of the Results and the "Genome sequencing and assembly" section of the Methods.

Suggest the authors to draw a figure to show the random genome editing pipeline more clear.
Answer: We have drawn a figure to show the random genome editing pipeline, please see the Additional File 8 in the revised manuscript.

'Evolutionary analysis of the genome' should be 'Evolutionary analysis of the genomes'
Answer: We have modified it according to your suggestion. Please see the title lines on page 4 and 10.

'the 351 individuals of the control group (wild-type) all died' remove the second 'all'.
Answer: We have modified it according to your suggestion. Please see the 3rd paragraph on page 5.

'The cumulative mortality curves was drawn' should 'were drawn'.
Answer: We have modified it according to your suggestion. Please see the 1st paragraph on page 12.

'Three wild-type females' should be 'Three wild-type female'.
Answer: We have modified it according to your suggestion. Please see the 2nd paragraph on page 12.

'in the same position in C.' changes to 'in the same position in C group'.
Answer: We have modified it according to your suggestion. Please see the 2nd paragraph on page 6.

All the software should be added the version and RRID in Gigascience journal.
Answer: Thank you and we have added the RRIDs of all software used in our manuscript.

Close
