# Supplementary material for: De novo screening of disease-resistant genes from the chromosome-level genome of rare minnow using CRISPR-cas9 random mutation
Source: Gigascience. 2021 Nov 19;10(11):giab075. doi: 10.1093/gigascience/giab075 (PMC8782236; doi:10.1093/gigascience/giab075)
Supplement: giab075_Response_to_Reviewer_Comments_Revision_1 [file giab075_response_to_reviewer_comments_revision_1.pdf]

Author's Response To Reviewer Comments

Close

Dear Mr. Wang,

Your revised manuscript "De novo screening of disease-resistant genes from the chromosome-level genome of rare minnow using CRISPR-cas9 random mutation" (GIGA-D-21-00147R1) has been re-assessed by two previous reviewers. I am pleased to inform you that it is potentially acceptable for publication in GigaScience, in principle, once you have carried out some minor follow-up revisions:
1) Please address reviewer #3 's comment about the phylogenetic ztree (see below this email).
Answer: Thank you for your reminder. We have redrawn the evolutionary tree by adding lamprey as an outgroup. Please see the Fig. 1b in the revised manuscript.

2) In your BUSCO analysis, you are using the general vertebrate gene set. Please re-do this analysis with the Actinopterygii gene set, and update the BUSCO results in the manuscript and files in your data sets
Answer: We have re-do this analysis with the Actinopterygii gene set, and updated the BUSCO results in the manuscript and files in the data sets. Please see the red font section of the revised manuscript and the data sets uploaded via FTP.

3) Prior to acceptance, our data curators will have another look at the data and they may have additional queries regarding the data files.
Answer: OK, please feel free to contact us.

Please also ensure that your revised manuscript conforms to the journal style, which can be found in the Instructions for Authors on the journal homepage. If the data and code has been modified in the revision process please be sure to update the public versions of this too.
The due date for submitting the revised version of your article is 28 Dec 2021.
We look forward to receiving your revised manuscript soon.

Best wishes,
Hans Zauner
GigaScience

Reviewer reports:
Reviewer #1: Thanks for your appropriate revisions and the additional viral infectivity experiment.
Answer: Thank you for your careful review.

Reviewer #3: The authors have well addressed my questions. The revision is pretty well.
An additional minor revision should be performed. In the divergence time tree of Figure 1, the placement of this tree is really incorrect. Why the frog branch is fused in the branch of teleost and tetropods?
Answer: Thank you for your valuable suggestions. What we drew before was an unrooted phylogenetic tree. In the revised manuscript, we added lamprey as an outgroup and redrawn a rooted phylogenetic tree, as shown in Fig. 1b. Please see the red font section of the revised manuscript and the data sets uploaded via FTP.

Close
