# Supplementary material for: De novo screening of disease-resistant genes from the chromosome-level genome of rare minnow using CRISPR-cas9 random mutation
Source: Gigascience. 2021 Nov 19;10(11):giab075. doi: 10.1093/gigascience/giab075 (PMC8782236; doi:10.1093/gigascience/giab075)
Supplement: giab075_Reviewer_1_Report_Original_Submission — Yehwa Jin -- 6/24/2021 Reviewed [file giab075_reviewer_1_report_original_submission.pdf]

Reviewer Report

Title: De novo screening of disease-resistant genes from the chromosome-level genome of rare minnow using CRISPR-cas9 random mutation

Version: Original Submission **Date:** 6/24/2021

Reviewer name: Yehwa Jin

Reviewer Comments to Author:

In this study, "De novo screening of disease-resistant genes at the chromosome-level genome of rare minnow using CRISPR/cas9 random mutation" Luo et al. reported a chromosome-level genome of wild-type rare minnow and a new approach of mutagenesis using universal guide RNAs for CRISPR-Cas9. Compared to genome-wide CRISPR knockout (GeCKO) screening, this approach does not require species-specific guide RNA libraries as the universal gRNA are designed to target all possible promoters or start codon regions. It demonstrated that candidate genes for disease resistance can be found using this approach. While this study presents a novel and useful approach of de novo screening of disease-resistant genes, I found some aspects that could be improved. In page 7, in the 3rd paragraph of the Discussion, '..however, the disadvantage is that it requires sufficient candidate gene sequences.', it is true but the method in the paper also requires the whole genome sequence information to analyse the mutations (although it is not required to design gRNAs in your method). In page 7, in the 4th paragraph of the Discussion, could you clarify what the mutation efficiency means? For example, when you say 0.23% mutation efficiency in rare minnow does it means 0.0023 X 943.44M bp = 2.17 M bp has been mutated in individual fish? In page 12, in the 3rd paragraph, could you describe the criteria for the four levels of contribution to gene function changes? In figure 2 d, e & f, could you consider making X-axis aligned among fig. 2 d, e & f? For example, make all X axis to have 15 days post infection as maximum value or 20 dpi.In figure 3, what does arrow means? Could you consider improve the figure 3 and the text in the 2nd paragraph in page 6 ('There were 147,679 (139632 + 1377 + …') for better readability? The viral loads were around 2 fold reduced in the knock-down (KD) grass carp cells for 7 target genes. However, 2 fold reduction in viral copy numbers is considered to be a subtle change and more importantly the viral load cannot confirm whether the viral infectivity was reduced. Analysing the infectivity of the virus produced from the KD cells by TCID50 or plaque assay together with the viral load is recommended. Regarding the viral load experiment, have you checked the viral load in the resistant and susceptible minnow in vivo? If so, was there a difference in viral load between the resistant and susceptible fish? I'm wondering why did you knock down the targets in grass carp cells instead of knockout? Knockout would be more suitable to validate the function of your candidate genes.Could you delineate the limitation of this approach in the discussion? For examples, for species with long generation time or screening for quantitative traits, this may not be feasible and etc. gRNAs were designed to target all protein coding genes but as the gRNA quantity for each target region is extremely low, so I think the mutations most likely happen to 'easy to target loci' which could leave numerous protein coding genes unmutated. Did you check how many genes were mutated in the founder fish before the viral challenge (to check the genome-wide mutation coverage of this 26 gRNA system)? Typos

or minor changes:P6, 2nd paragraph, '..(inner ring of Fi. 3).' to be '..(inner ring of Fig. 3).'P12, 3rd paragraph, '…without a typing result..' to be '…without a genotyping result..'.P13, 2nd paragraph, 'Genes with inhibitory effects…' to be 'siRNAs with inhibitory effects…'.P3, 3rd paragraph, please consider use 'CRISPR-Cas9' instead of 'CRISPR/Cas9' as '-' means 'and' while '/' means 'or'.

Level of Interest

Please indicate how interesting you found the manuscript: Choose an item.

Quality of Written English

Please indicate the quality of language in the manuscript: Choose an item.

Declaration of Competing Interests

Please complete a declaration of competing interests, considering the following questions:

- Have you in the past five years received reimbursements, fees, funding, or salary from an organisation that may in any way gain or lose financially from the publication of this manuscript, either now or in the future?
- Do you hold any stocks or shares in an organisation that may in any way gain or lose financially from the publication of this manuscript, either now or in the future?
- Do you hold or are you currently applying for any patents relating to the content of the manuscript?
- Have you received reimbursements, fees, funding, or salary from an organization that holds or has applied for patents relating to the content of the manuscript?
- Do you have any other financial competing interests?
- Do you have any non-financial competing interests in relation to this paper?

If you can answer no to all of the above, write 'I declare that I have no competing interests' below. If your reply is yes to any, please give details below.

I declare that I have no competing interests.

I agree to the open peer review policy of the journal. I understand that my name will be included on my report to the authors and, if the manuscript is accepted for publication, my named report including any attachments I upload will be posted on the website along with the authors' responses. I agree for my report to be made available under an Open Access Creative Commons CC-BY license (http://creativecommons.org/licenses/by/4.0/). I understand that any comments which I do not wish to be included in my named report can be included as confidential comments to the editors, which will not be published.

Choose an item.

To further support our reviewers, we have joined with Publons, where you can gain additional credit to further highlight your hard work (see: https://publons.com/journal/530/gigascience). On publication of this paper, your review will be automatically added to Publons, you can then choose whether or not to claim your Publons credit. I understand this statement.

Yes Choose an item.
