# Supplementary material for: De novo screening of disease-resistant genes from the chromosome-level genome of rare minnow using CRISPR-cas9 random mutation
Source: Gigascience. 2021 Nov 19;10(11):giab075. doi: 10.1093/gigascience/giab075 (PMC8782236; doi:10.1093/gigascience/giab075)
Supplement: giab075_Reviewer_2_Report_Original_Submission — Dietmar KÃ¼ltz -- 7/9/2021 Reviewed [file giab075_reviewer_2_report_original_submission.pdf]

Reviewer Report

Title: De novo screening of disease-resistant genes from the chromosome-level genome of rare minnow using CRISPR-cas9 random mutation

Version: Original Submission **Date:** 7/9/2021

Reviewer name: Dietmar KÃ¼ltz

Reviewer Comments to Author:

This is an interesting experiment using an innovative combination of random CRISPR/Cas9 mutagenesis with GCRV infection as a powerful selection tool to screen for disease relevant genes. Because of such a high degree of degeneracy in the gRNA sequences, it seems unlikely there would be bi-allelic mutations of any given gene in the injected embryos and thus the method would only find genes in which there is a mono-allelic affect. For the same reason, I was initially concerned that the GCRV survival phenotype would be a result from a combination of so many differ individual mutations making it difficult to isolate individual relevant genes. However, it does seem the screening methods described are sufficient to find at least some GCRV relevant genes, and thus this work is definitely useful and very worthwhile to report. I do have a few concerns and questions I hope can be addressed. I am somewhat skeptical there was value targeting the TATA box considering only ~20% of promoters contain this element. Was it recorded what percentage of the 23 hemorrhagic disease associated loci came from TATAWAW targeting gRNAs in contrast to ATG targeting gRNAs? This information should be included and discussed in the manuscript. In the "Functional verification of susceptible genes related to hemorrhagic disease" section of the results, the 23 candidate genes were narrowed down to 9. The wording was not clear, you wrote "This indicated that the siRNA of the nine genes had a significant inhibitory effect". I'm assuming you meant, This indicated that nine siRNAs had a significant inhibitory effect". In the methods section, it is not clear why every gRNA sequence was started with an NNR. This should be clarified. As far as functional validation, it seems micro-injection of single gene specific gRNAs targeting the 23 candidate genes from the initial screen would have been most appropriate and should be done in addition to or in place of the siRNA treated GCO cell line validation. What was the rationale behind taking only this latter approach? This rationale should be outlined in the manuscript to better justify the experimental design.

Level of Interest

Please indicate how interesting you found the manuscript: Choose an item.

Quality of Written English

Please indicate the quality of language in the manuscript: Choose an item.

Declaration of Competing Interests

Please complete a declaration of competing interests, considering the following questions:

- Have you in the past five years received reimbursements, fees, funding, or salary from an organisation that may in any way gain or lose financially from the publication of this manuscript, either now or in the future?
- Do you hold any stocks or shares in an organisation that may in any way gain or lose financially from the publication of this manuscript, either now or in the future?
- Do you hold or are you currently applying for any patents relating to the content of the manuscript?
- Have you received reimbursements, fees, funding, or salary from an organization that holds or has applied for patents relating to the content of the manuscript?
- Do you have any other financial competing interests?
- Do you have any non-financial competing interests in relation to this paper?

If you can answer no to all of the above, write 'I declare that I have no competing interests' below. If your reply is yes to any, please give details below.

I declare that I have no competing interests

I agree to the open peer review policy of the journal. I understand that my name will be included on my report to the authors and, if the manuscript is accepted for publication, my named report including any attachments I upload will be posted on the website along with the authors' responses. I agree for my report to be made available under an Open Access Creative Commons CC-BY license (http://creativecommons.org/licenses/by/4.0/). I understand that any comments which I do not wish to be included in my named report can be included as confidential comments to the editors, which will not be published.

Choose an item.

To further support our reviewers, we have joined with Publons, where you can gain additional credit to further highlight your hard work (see: https://publons.com/journal/530/gigascience). On publication of this paper, your review will be automatically added to Publons, you can then choose whether or not to claim your Publons credit. I understand this statement.

Yes Choose an item.
