# Supplementary material for: De novo screening of disease-resistant genes from the chromosome-level genome of rare minnow using CRISPR-cas9 random mutation
Source: Gigascience. 2021 Nov 19;10(11):giab075. doi: 10.1093/gigascience/giab075 (PMC8782236; doi:10.1093/gigascience/giab075)
Supplement: giab075_Reviewer_3_Report_Original_Submission — Chao Bian -- 8/3/2021 Reviewed [file giab075_reviewer_3_report_original_submission.pdf]

Reviewer Report

Title: De novo screening of disease-resistant genes from the chromosome-level genome of rare minnow using CRISPR-cas9 random mutation

Version: Original Submission **Date:** 8/3/2021

Reviewer name: Chao Bian

Reviewer Comments to Author:

This paper entitled as 'De novo screening of disease-resistant genes at the chromosome-level genome of rare minnow using CRISPR/cas9 random mutation' has shown a novel chromosome-level genome of rare minnow. The authors performed a series of analyses, including genome assembly, annotation, evolution and gene editing. The methods used in this paper are really solid and common.However, the most significant detect is why this fish is a better gene editing model than zebrafish, which is not clearly described in this study. On the other hand, the genome assembly has not been evaluated by BUSCO.The English language of this paper needs a large of revision.In Abstract, the assembled chromosome number should be clarified in this section.In Introduction, 'It is worth noting that they are more sensitive to organic pollutants and pathogenic microorganisms than zebrafish (Danio rerio) and medaka (Oryzias latipes).' This sentence should be more clarified why this species is more sensitive. The related reference also should be complemented in this sentence."Traditional physical and chemical mutagenesis methods mainly cause genomic point mutations [4-6], which cannot be distinguished from natural SNP mutations, which leads to considerable difficulties while performing comparative analysis of the subsequent functional genomes." This sentence should be revised, two 'which' are indeed strange.'The traditional transposon mutation has' revised to 'The traditional transposon mutations have'.'On one hand, the cost of designing a large number of sgRNAs is high; on the other hand, it is only suitable for the construction of a mutant library of known candidate genes.' should be revised.'Therefore, the genome of the rare minnow was deemed to be a simple one.' What's real meaning of the 'simple one'?'the N50 was 21,231 bp' should be keep two decimals.'181,735,272 pairs of unique mapped read pairs were obtained' revise to '181,735,272 pairs of unique mapped reads were obtained'.'a total of 36,387 mRNA encoding proteins were annotated, corresponding to 26,861 genes.' Why 26,861 genes were related to 36387 mRNA? This set contains alternative splicing results? No BUSCO evaluation result for the genome assembly.Suggest the authors to draw a figure to show the random genome editing pipeline more clear. 'Evolutionary analysis of the genome' should be 'Evolutionary analysis of the genomes''the 351 individuals of the control group (wild-type) all died' remove the second 'all'.'The cumulative mortality curves was drawn' should 'were drawn'.'Three wild-type females' should be 'Three wild-type female'.'in the same position in C.' changes to 'in the same position in C group'.All the software should be added the version and RRID in Gigascience journal.

Level of Interest

Please indicate how interesting you found the manuscript: Choose an item.

Quality of Written English

Please indicate the quality of language in the manuscript: Choose an item.

Declaration of Competing Interests

Please complete a declaration of competing interests, considering the following questions:

- Have you in the past five years received reimbursements, fees, funding, or salary from an organisation that may in any way gain or lose financially from the publication of this manuscript, either now or in the future?
- Do you hold any stocks or shares in an organisation that may in any way gain or lose financially from the publication of this manuscript, either now or in the future?
- Do you hold or are you currently applying for any patents relating to the content of the manuscript?
- Have you received reimbursements, fees, funding, or salary from an organization that holds or has applied for patents relating to the content of the manuscript?
- Do you have any other financial competing interests?
- Do you have any non-financial competing interests in relation to this paper?

If you can answer no to all of the above, write 'I declare that I have no competing interests' below. If your reply is yes to any, please give details below.

no

I agree to the open peer review policy of the journal. I understand that my name will be included on my report to the authors and, if the manuscript is accepted for publication, my named report including any attachments I upload will be posted on the website along with the authors' responses. I agree for my report to be made available under an Open Access Creative Commons CC-BY license (http://creativecommons.org/licenses/by/4.0/). I understand that any comments which I do not wish to be included in my named report can be included as confidential comments to the editors, which will not be published.

Choose an item.

To further support our reviewers, we have joined with Publons, where you can gain additional credit to further highlight your hard work (see: https://publons.com/journal/530/gigascience). On publication of this paper, your review will be automatically added to Publons, you can then choose whether or not to claim your Publons credit. I understand this statement.

Yes Choose an item.
